# Comparative Genomics Reveals Multiple Genetic Backgrounds of Human Pathogenicity in the *Trypanosoma brucei* Complex

**DOI:** 10.1093/gbe/evu222

**Published:** 2014-10-05

**Authors:** Mark Sistrom, Benjamin Evans, Robert Bjornson, Wendy Gibson, Oliver Balmer, Pascal Mäser, Serap Aksoy, Adalgisa Caccone

**Affiliations:** ^1^Department of Ecology and Evolutionary Biology, Yale University; ^2^Department of Computer Science, Yale University; ^3^School of Biological Sciences, University of Bristol, United Kingdom; ^4^Swiss Tropical and Public Health Institute, Basel, Switzerland; ^5^Zoological Institute, University of Basel, Switzerland; ^6^Department of Epidemiology of Microbial Diseases, Yale School of Public Health, New Haven, CT

**Keywords:** comparative genomics, genomics, trypanosomatids, next gen sequencing, population genomics

## Abstract

The *Trypanosoma brucei* complex contains a number of subspecies with exceptionally variable life histories, including zoonotic subspecies, which are causative agents of human African trypanosomiasis (HAT) in sub-Saharan Africa. Paradoxically, genomic variation between taxa is extremely low. We analyzed the whole-genome sequences of 39 isolates across the *T. brucei* complex from diverse hosts and regions, identifying 608,501 single nucleotide polymorphisms that represent 2.33% of the nuclear genome. We show that human pathogenicity occurs across a wide range of parasite genotypes, and taxonomic designation does not reflect genetic variation across the group, as previous studies have suggested based on a small number of genes. This genome-wide study allowed the identification of significant host and geographic location associations. Strong purifying selection was detected in genomic regions associated with cytoskeleton structure, and regulatory genes associated with antigenic variation, suggesting conservation of these regions in African trypanosomes. In agreement with expectations drawn from meiotic reciprocal recombination, differences in average linkage disequilibrium between chromosomes in *T. brucei* correlate positively with chromosome size. In addition to insights into the life history of a diverse group of eukaryotic parasites, the documentation of genomic variation across the *T. brucei* complex and its association with specific hosts and geographic localities will aid in the development of comprehensive monitoring tools crucial to the proposed elimination of HAT by 2020, and on a shorter term, for monitoring the feared merger between the two human infective parasites, *T. brucei rhodesiense* and *T. b. gambiense,* in northern Uganda.

## Background

Recent advances in DNA sequencing technology provide an unprecedented ability to investigate the genomic variation of human pathogens to understand their pathogenicity and evolutionary history with the goal of improving drug and vaccine design. Comparative analysis of variation at the genome level is a crucial step to understand the variation in pathogen populations. However, the large genome size of most eukaryotic pathogens has limited our ability to generate comprehensive and comparative genomic data to date.

Within the genus *Trypanosoma* (*Kinetoplastida*), *Trypanosoma brucei rhodesiense* (*Tbr*) and *Trypanosoma brucei gambiense* (*Tbg*) are the causative agents of human African trypanosomiasis (HAT) (or sleeping sickness), whereas *T. b. brucei* (*Tbb*), *Trypanosoma congolense*, and *Trypanosoma vivax* (*Tv*) cause Animal African Trypanosomiasis (AAT or Nagana). African trypanosomiasis impacts both human and animal health in sub-Saharan Africa (Simarro, Cecchi, et al. 2012). In 2008, mortality associated with HAT ranked ninth out of 25 among the human infectious and parasitic diseases in Africa (Fèvre et al. 2008). *Tbg* causes a chronic disease with asymptomatic periods lasting several years, whereas *Tbr* causes an acute disease with over 80% mortality within the first 6 months if untreated. Over 90% of the HAT cases reported are due to *Tbg* occurring in northwest Uganda, extending into the Central African Republic and to Equatorial Guinea. Over 12 million people in eastern and southern Africa, including Uganda, Tanzania, Malawi, Zambia, and Zimbabwe are at risk for *Tbr* (Simarro, Cecchi, et al. 2012). Intense international interventions recently reduced HAT cases below 10,000 for the first time in 50 years (Simarro et al. 2011), but many cases likely go undetected in remote regions (Simarro et al. 2011). Available drugs for treatment are expensive, associated with adverse effects (Simarro, Franco, et al. 2012), and exhibit increasing drug resistance (Brun et al. 2001). Thus, there are significant challenges to the proposed elimination of HAT by 2020 (WHO 2012). Uganda is the only country in Africa where the two HAT causing parasites (*Tbg* and *Tbr*) occur. They are currently less than 100 km apart, with the *Tbr* distribution expanding westwards toward *Tbg* endemic areas (Picozzi et al. 2005). As the pathology, diagnosis, and treatment vary between the two disease forms, an overlap of the two disease belts is feared to complicate HAT control (Welburn et al. 2009).

The relationships between taxa in the *T. brucei* complex are incompletely understood (Koffi et al. 2009; Balmer et al. 2011), hampering the development of effective disease controls. The current subdivision of *T. brucei* into three subspecies (*Tbb*, *Tbr*, and *Tbg*) is based on host range, pathogenicity, and geographic origin, and does not reflect genetic or evolutionary distinctions (Gibson 1986; Balmer et al. 2011). *Tbb* occurs across sub-Saharan Africa whereas *Tbr* and *Tbg* are distributed allopatrically in eastern/southern Africa and in western/central Africa, respectively. *Tbg* is further subdivided into *Tbg* group 1 (*Tbg1*), responsible for greater than 90% of HAT cases in Africa, and a minor type, *Tbg* group 2 (*Tbg2*), represented by a single known laboratory strain (Gibson 1986).

The diagnostic feature of *Tbr* is the presence of the serum resistance associated (SRA) gene (Gibson 2005), which enables *Tbr* to evade lysis by the *Apolipoprotein L-1* (*ApoL-1*) in human serum (Van Xong et al. 1998). Aside from this crucial functional distinction, previous studies, including most recently a kinetoplast *CO1* (*Cytochrome Oxidase*) sequence and eight nuclear microsatellites of 142 isolates across Africa (Balmer et al. 2011), have shown lack of differentiation between *Tbb* and *Tbr*, while finding clear genetic differences between *Tbg* and *Tbb/Tbr* (Gibson 2005). Neither the genome of *Tbg1* nor *Tbg2* has the *SRA* gene, but both parasites have evolved independent mechanisms for evading trypanolysis by *ApoL-1* (Capewell et al. 2011; Jackson et al. 2012). A recent population survey found this mechanism to be associated with a single functionally relevant nonsynonymous substitution in the haptoglobin–hemogolobin receptor gene (Symula et al. 2012; DeJesus et al. 2013). *Trypanosoma evansi* (*Te*), although described as a different species, is regarded as a *Tbb* mutant form (Lai et al. 2008), which has become an important livestock pathogen causing “surra” across subtropical regions across the world, having acquired the ability of being transmitted by biting flies other than tsetse.

The nuclear genome of *T. brucei* comprised 11 chromosomes totaling 26 Mb in size, making whole-genome sequencing (WGS) of multiple individuals increasingly tractable. Although assembled reference genomes for two *T. brucei* subspecies—*Tbb* (Berriman et al. 2005) and *Tbg1* (Jackson et al. 2010)—are available, *T. brucei* poses a major challenge to comparative genomic studies as up to 30% of the genome includes subtelomeric regions largely made up of repetitive pseudogene arrays, which are associated with antigenic variation (Berriman et al. 2005). In addition to these subtelomeric repeat arrays, *T. brucei* has a variable number of small- to intermediate-sized chromosomes (30–700 kb) (Berriman et al. 2005; Jackson et al. 2010), encoding sequences similar to the subtelomeric regions of the larger megabase chromosomes (Bringaud et al. 2002).

Previous studies have shown that despite the considerable variation in life history traits and clinical disease outcome, genomic variation is extremely limited with less than 1% sequence divergence observed between *Tbb* and *Tbg1* coding regions (Jackson et al. 2010). This limited genomic variation suggests that the observed functional differences could be due to features shared between subspecies, and vary in either structure or expression (Jackson et al. 2010). Thus, an important step for assessing the genomic basis of clinical disease types and life history differentiation in *T. brucei* is to determine levels and patterns of standing genomic variation within and between these taxa.

In this study, we obtained WGS of 39 isolates representing all three endemic African *T. brucei* named subspecies and *Te.* These isolates encompass a wide range of geographic localities, hosts, and dates of initial collection (supplementary appendix S1, Supplementary Material online). Using the published TREU927/4 *Tbb* reference genome (Berriman et al. 2005), we identified single nucleotide polymorphism (SNP) positions, determined genomic variation and admixture between strains, and looked for signatures of selection and linkage across the *T. brucei* genome. We used the results of these analyses to evaluate the evolutionary processes shaping genomic variation and explore the utility of comparative genomics in both the development of HAT treatments and as monitoring and control tools.

## Materials and Methods

### Sample Preparation, Sequencing, and Quality Control

Supplementary appendix S1, Supplementary Material online, lists the cloned strains analyzed in this study, which include 19 *Tbb*, 13 *Tbr*, 2 *Tbg*1, 1 *Tbg2*, and 4 *Te* strains, together with country of origin and host. DNA was extracted from cryopreserved isolates from the Swiss Tropical and Public Health Institute, Basel or the University of Bristol. All strains were isolated in previous studies in adherence with national and institutional guidelines and extractions carried out using either a Qiagen micro DNA kit (Qiagen Pty Ltd) or using standard phenol–chloroform protocols dependent on sample quality. Fragmentation and library preparation and sequencing (2 × 75 bp) were conducted at the Yale Center for Genome Analysis, using either an Illumina Genome Analyzer IIx platform (STIB809) or an Illumina HiSeq 2000 platform (all other isolates) (data will be submitted to the National Center for Biotechnology Information short sequence read database upon acceptance). Quality control of reads was conducted using FastQC (Andrews 2010).

### Read Mapping and SNP Site Calling

Paired-end reads were mapped to the publicly available version of the *Tbb* TREU927/4 reference genome (Berriman et al. 2005) (updated September 2011), using BWA 0.6.2-r126 (Li and Durbin 2009) with the default parameters. Improperly paired reads were not used for SNP calling. Read coverage after filtering was visualized using Circos (Krzywinski et al. 2009). SNP calling was conducted using the Genome Analysis Toolkit (GATK v2.2-15) (McKenna et al. 2010), with the minimum PHRED-scaled variant confidence set to 30. The raw calls were further manipulated in variant call format with VCFtools (v0.1.10) (Danecek et al. 2011). Individual genotypes with depth of coverage less than 10 and/or a quality score of less than 20 were discarded, as well as SNP positions with a quality score less than 50. SNP density was visualized using Circos (Krzywinski et al. 2009). To evaluate our ability to accurately map reads to the reference genome, we calculated repetition scores across the genome. To do this, we fragmented the genome into 32-bp segments and calculated how many times a given 32-bp sequence occurred throughout the genome, using a custom Python script. We selected 32 bp as this is slightly less than half of an Illumina read and thus a conservative measure that should identify regions where a full read should be able to identify a unique gene sequence. Repetition scores were averaged across 10-kb windows, visualized using Circos (Krzywinski et al. 2009), and overlaid on average coverage.

### Whole-Genome Variation between Isolates

Using a custom Python script we attempted to identify diagnostic SNP between the named subspecies (SNP positions, which were fixed within subspecies, but different between). For the analysis, we excluded sites with missing data from any individual. SNP data were then converted to PLINK format for further analysis, using the Adegenet package (Jombart 2008) in the R statistical environment (R Core Team 2011). The program Admixture (Alexander et al*.* 2009) was used to evaluate whole-genome SNP variance among strains using. We explored *K* values from 1 to 25 assessed convergence by studying the Log-likelihood scores (LLs). We note that for all values of *K*, the maximum difference of LLs within the fraction of runs (5) yielding the highest LLs was minimal (up to 0.14 LL units). Though not definitive, this observation is indicative that convergence was reached for all values of *K* tested.

### Selection and Linkage Disequilibrium

In order to investigate selection across *Tbb*/*Tbr*/*Tbg2*, we calculated Tajima’s *D* (Tajima 1989) across the genome for the 33 *Tbb*/*Tbr*/*Tbg2* samples using VCFtools v0.1.10 (Danecek et al. 2011) across the 11 Mb chromosomes. We investigated both SNP density and Tajima’s *D* at a range of nonoverlapping window sizes (see supplementary material, Supplementary Material online) and selected 10-kb windows as a balance of both resolution and accuracy. Regions under strong selection were determined by separating out windows with positive and negative Tajima’s *D* values. The lowest 5% of negative values (potentially indicative of purifying selection) and the highest 5% of positive values (potentially indicative of balancing or diversifying selection) were defined as being under strong selection. These windows were plotted using Circos (Krzywinski et al. 2009). To confirm selection on genes within windows identified as under strong selection, we calculated Tajima’s *D* for all annotated genes within these windows using VCFtools (Danecek et al. 2011). Additionally, we performed a McDonald–Kreitman (MK) test (McDonald and Kreitman 1991) for all the 9,068 annotated genes in the Treu927 genome (Berriman et al. 2005). Each alignment was BLAST searched against the *Tv* genome to add an outgroup sequence, using a minimum *e* value of 10^−^^3^ and a maximum of three hits. Subsequent alignment was undertaken using MUSCLE v 3.8.31 (Edgar 2004) and MK tests for each alignment were conducted using the R package (R Core Team 2011) Popgenome (Pfeifer et al. 2014). To further explore the function of genes putatively under selection, we extracted gene ontology (GO) terms using AmiGO 2 (Carbon et al. 2009). These results were further refined by determining GO terms significantly enriched in both positively and negatively selected gene sets. Enriched GO terms were determined using a minimum *P* value of 0.01 and a minimum of two genes for each term.

To evaluate linkage disequilibrium (LD), exhaustive intrachromosomal pairwise calculations of the disequilibrium coefficient, *r*^2^, were computed between SNPs within each chromosome, using the program PLINK v1.07 (Purcell et al. 2007). To determine the distance of physical linkage for each chromosome, we plotted the average *r*^2^ against the distances between SNPs for distances between 1 and 100,000 bp and fitted a Loess nonparametric regression line to each chromosome. To evaluate potential causes of differing linkage in each chromosome we performed a multiple regression with r2 using the base package of R (R Core Team 2011) as the dependent variable, and chromosome length, number of annotated repeat regions, and number of annotated variant surface glycoprotein (VSG) genes after eliminating comparisons over distances greater than 25,000 bp to ensure that physical linkage did not confound results. This was followed by k-fold cross validation (CV) in the R package DAAG (Maindonald and Braun 2006) to evaluate the relationships between these factors and linkage.

## Results

### Sequencing, Alignment, SNP Calling, and Repetition Analysis

**Fig. 1.—:**
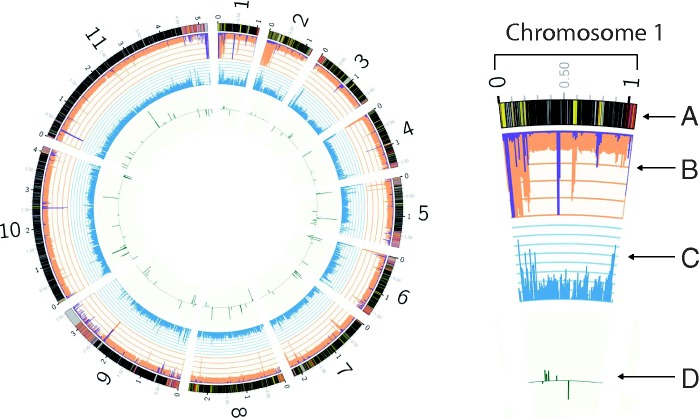
Results from comparative genomic analyses. Chromosome 1 is shown in the upper right inset to highlight details on the overall figure. Chromosomes are numbered (large numbers, 1–10, at the outer edge of the circle) corresponding to the TREU927/4 genome annotation. Data tracks are listed from outermost to innermost in the inset, small black and gray numbers indicate positions in megabases. Track A: A summary of coding (black), noncoding (gray) regions, annotated repeat regions (yellow), and annotated VSG (red) genes. Track B: Coverage (orange) overlaid with repetition (purple) averaged in 10-kb windows. Scale lines represent 100× average coverage/repetition per window. Track C: SNP density summarized as average number of SNPs per kilobase in 10-kb windows. Scale lines represent ten SNPs per kilobase. Track D: Windows under high levels of selection as determined by calculations of Tajima’s *D*. Green bars indicate windows in which the Tajima’s *D* score was in the highest and lowest 5% of values above and below zero.

We recovered an average of 76.7 million reads per isolate, on average 73% aligned to the reference TREU927/4 genome (139× average coverage/isolate). The relatively high number of unmapped reads is likely to be explained by the fact that we did not include the highly repetitive kinteoplast minichromosomes in our reference, which make up a significant proportion of the *T. brucei* genome (Berriman et al. 2005). Initial SNP calling identified approximately 1.9 million SNP sites (∼1.5 million in the 11 Mb chromosomes). Quality filtering and the exclusion of SNPs with missing data for at least one isolate reduced the data set to 608,501 SNPs (fig. 1). Analysis of repetitive genomic regions (fig. 1) showed high genomic repetitiveness in VSG regions and annotated repeats, in addition to a number of unannotated regions (e.g., the end of chromosome 9). As we excluded all SNPs in these regions from further analyses, the final SNP data set included 579, 129 SNPs.

### Genomic Variation and Clustering

**Fig. 2.—:**
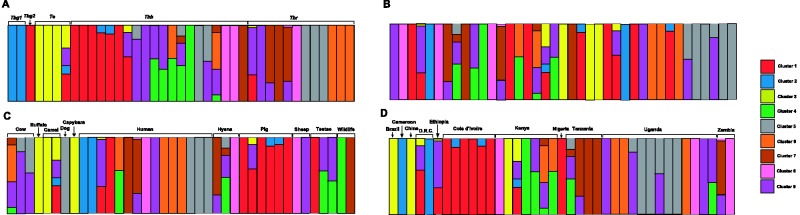
Cluster diagram of individual assignment of each isolate to the nine clusters inferred using Admixture (Alexander et al. 2009). Bars represent individuals and colors indicate each genetic cluster inferred with greater than 0.95 support. The height of each bar on the left indicates the probability with which each isolate was assigned to each cluster. Isolates are arranged by (*A*) subspecies, (*B*) date of isolation, (*C*) host species, and (*D*) country of isolation. Details for each strain are listed in supplementary appendix S1, Supplementary Material online.

Results from Admixture (Alexander et al. 2009) showed that CV error was lowest when *K* = 9 (supplementary fig. S4, Supplementary Material online) although similar levels of CV error were observed from *K* = 5–9. Cluster 2 includes the *Tbg1* samples and cluster 3 *Te* samples. Isolates from *Tbg2*, *Tbb,* and *Tbr* are interspersed across the other seven clusters. Admixture analysis (Alexander et al*.* 2009) to determine the assignment probability of each isolate to the nine clusters revealed substantial admixture between clusters (fig. 2). Chi-square analysis (table 1) showed that both geography and host significantly account for cluster assignment. In particular, clusters 4 and 5 in figure 2 group all seven *Tbb* isolates from West/Central African livestock, humans, and tsetse. In contrast, *Tbb* isolates from East Africa are spread between clusters 6 and 9. We found a significant correlation between cluster assignment and both host and country of collection, but did not find a correlation between cluster assignment and collection date or subspecies assignment.

**Table 1:** Results of χ^2^ Tests to Determine Significant Correlations between Genomic Clustering and Subspecies Designation, Host Species, Date of Collection and Country of Collection

Test Parameter	χ^2^	df	*P* Value
Subspecies	9.38	10	0.5
Country	129.17	40	<0.01*
Host	124.87	40	<0.01*
Date collected	101.61	95	0.3

Note.—df, degrees of freedom,

*Significant values.

### Genomic Patterns of Selection and LD

Investigating selection across *Tbb*/*Tbr*/*Tbg2* by calculating Tajima’s *D* (Tajima 1989) in 10-kb windows revealed that much of the *T. brucei* genome is under purifying selection. We found 43 windows (i.e., 430 kb) in the lowest 5% of Tajima’s *D* values and therefore under strong purifying selection. These windows contain 1,290 annotations—which in turn are largely conserved across Tbb/Tbr/Tbg2, although 69.6% are annotated as hypothetical coding regions or miscellaneous annotations yet to be characterized. Of the genes of known function, 58% encode proteins common to the majority of all eukaryotes, an expected result, which also validates our methodology.

We found 80 windows (i.e., 800 kb) in the lowest 5% of Tajima’s *D* values and therefore under strong diversifying selection. These windows contain 2,180 annotations, of which 60.4% are annotated as hypothetical coding regions or miscellaneous annotations yet to be characterized (fig. 1 and supplementary appendix S2, Supplementary Material online). Tajima’s *D* was calculated for each annotation in the strongly selected windows and is shown in supplementary appendix S2, Supplementary Material online. As expected, many of the genes in windows under strong purifying selection had no variation and thus calculating Tajima’s *D* was not possible. However, lack of variation in these genes is in itself a strong indicator of their conservation across *T. brucei.* Selection was also estimated using the MK test on a total of 2,889 annotated genes. However, due to a large number of gaps in the alignment with the *Tv* outgroup sequence, which are incorrectly interpreted as nonsynonymous mutations by the MK test, most alignments resulted in infinite values. Nevertheless, values were obtained for 208 genes (supplementary appendix S3, Supplementary Material online). Crossreferencing this list with the genes identified using Tajima’s *D* revealed that only three genes were able to have selection coefficients calculated using both MK and Tajima’s *D* tests (supplementary appendix S2, Supplementary Material online). Analyzing GO term enrichment showed that three terms were significantly enriched in positively and negatively selected regions (table 2). All of the terms significantly enriched in the purifying gene set related to cytoskeleton structure, and all of those significantly enriched in the diversifying gene set related to lysate activity.

**Table 2:** GO Terms Found to be Significantly Enriched in Genomic Windows under High Selection

GO Term	Aspect	*P* Value	Genes
Purifying selection			
GO:0009975 cyclase activity	F	>0.01	Tb927.5.320 Tb927.5.330 Tb927.7.6060 Tb927.10.2430
			Tb927.6.280 Tb927.7.6050 Tb927.4.3880 Tb927.6.300
			Tb927.9.14410
GO:0004016 adenylate cyclase activity	F	>0.01	Tb927.5.320 Tb927.5.330 Tb927.7.6060 Tb927.10.2430
			Tb927.6.280 Tb927.7.6050 Tb927.4.3880 Tb927.6.300
GO:0016849 phosphorus-oxygen lyase activity	F	>0.01	Tb927.5.320 Tb927.5.330 Tb927.7.6060 Tb927.10.2430
			Tb927.6.280 Tb927.7.6050 Tb927.4.3880 Tb927.6.300
Diversifying selection			
GO:0000226 microtubule cytoskeleton organization	P	>0.01	Tb927.1.2380 Tb927.1.2360 Tb927.1.2350 Tb927.1.2370
			Tb927.1.2330 Tb927.1.2340
GO:0005874 microtubule	C	>0.01	Tb927.1.2380 Tb927.1.2360 Tb927.1.2350 Tb927.1.2370
			Tb927.1.2330 Tb927.1.2340
GO:0005200 structural constituent of cytoskeleton	F	>0.01	Tb927.1.2380 Tb927.1.2360 Tb927.1.2350 Tb927.1.2370
			Tb927.1.2330 Tb927.1.2340

Note.—Aspect refers to the ontology aspect of the enriched term; C, cellular component; F, molecular function; P, biological process.

**Fig. 3.—:**
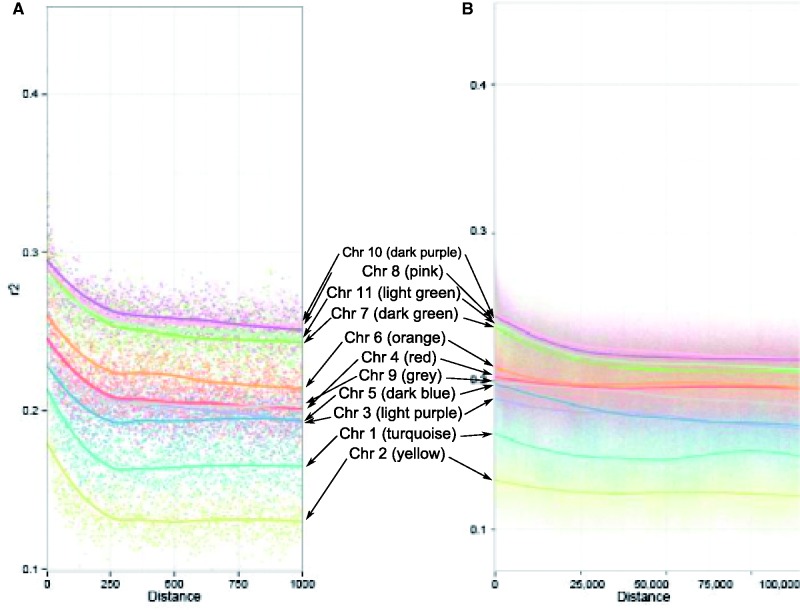
Plots of average linkage coefficient *r*^2^ scores against the distance between SNPs for each chromosome for distances between 1 and 1,000 bp (*A*) and between 1 and 100,000 bp (*B*). The average for value of *r*^2^ for each chromosome is plotted as a point, the fitted line represents a Loess nonparametric regression line of best fit to the average *r*^2^ for each value.

We evaluated LD by calculating the disequilibrium coefficient *r*^2^ between all SNPs in a pairwise fashion (Purcell et al. 2007) on each chromosome and plotted average *r*^2^ against physical distance for each chromosome (fig. 3). The regression lines fitted to each chromosome show that physical linkage decays in a similar pattern for all chromosomes (fig. 3). However, although the shape of *r*^2^ decay remains similar across chromosomes, the average *r*^2^ differs substantially between them (fig. 3). Although chromosomes with high numbers of annotated repeat regions and VSG genes could potentially result in inflated recombination rates and thus lower average *r*^2^ values, multiple linear regression analysis shows that, although a significant positive correlation between *r*^2^ and chromosome length is present, no correlation between *r*^2^ and the number of annotated repeat regions or VSG gene annotations could be detected (table 3).

**Table 3:** Results of Multiple Regression Analyses to Determine Significant Correlations between the Average *r*^2^ of Each Chromosome and Chromosome Length (Length), Number of Annotated Repeat Regions (Repeats), and Number of Annotated VSG Gene Regions (VSG)

Parameter	*r*^2^	Standard Error	*t* value
Overall	7.10E-02	2.50E-02	2.84
Length	2.20E-08	6.79E-09	3.25
Repeats	1.25E-05	1.70E-05	0.74
VSG	−1.11E-04	6.04E-05	−1.84

Note.—Scores are shown for all parameters combined (overall), in addition to the contribution of each.

## Discussion

### Sequencing, Alignment, SNP Calling, and Repetition Analysis

SNP density appears to be relatively uniform across the nuclear genome and most windows across the *T. brucei* genome were relatively unique and thus confidently mapped and SNP called (fig. 1). However, a small percentage of windows, especially in the highly repetitive subtelomeric regions containing VSG gene archives, which are critical for antigenic variation in *T. brucei* (Pays et al. 2004), had high numbers of reads mapped. This is indicative of mismapping of the short read sequences to these regions, which are known to be repetitive (Treangen and Salzberg 2012). These sites showed a steep decline in SNP density, demonstrating that quality filtering of SNP sites we used was effective (fig. 1). Although we excluded from downstream analyses all SNPs in these regions, as they have a high probability of being false positives, this resulted in the exclusion of only 4.8% of SNPs called, which is unlikely to have biased the interpretation of our results. Investigating the genetic diversity in these highly repetitive regions represents a challenge for all current sequencing and analysis methodologies (Treangen and Salzberg 2012), and it would require a de novo assembly of the *Tbr* genome, currently unavailable.

### Genomic Variation and Clustering

Figure 2 shows patterns of genomic variation across the 39 isolates analyzed. It is particularly striking that *Tbr* isolates, including several from the ongoing new HAT epidemic in central Uganda, are highly diverse, as they do group in different clusters. This supports the hypothesis that new *Tbr* strains are being generated by transfer of the SRA gene to new genetic backgrounds (Balmer et al. 2011). Figure 2 also provides genomic-scale support for the possibility that *Tbg2* is evolutionarily, as well as functionally, more closely allied to *Tbb* than *Tbg1* (Capewell et al. 2011), although this result bears caution, as it is based on the analysis of a single *Tbg2* isolate, the only one currently available in cryobanks.

We also show that the assignment of isolates to clusters is significantly explained by both country of origin and host species, demonstrating a strong geographic component that, together with host specificity, plays a role in shaping the genomic diversity of this species complex (table 1). As isolates do not cluster by subspecies, these findings support the previously stated hypothesis that differences in life history and disease type in *T. brucei* are due to variance in either the structure or expression of genes shared between subspecies, rather than fixed differences between them (Jackson et al. 2010). This also suggests that temporal turnover and the life history characteristics/clinical disease manifestation, which are used to define subspecies in *T. brucei*, do not explain genome-level differences between the sampled isolates.

The lack of any diagnostic SNPs between the named subspecies further underscores the importance of derived, functional elements that are either too repetitive or not present in the reference genome in the disparate life histories observed between *T. brucei* subspecies. Investigating subspecies-specific genomic regions could yield additional insights into the genomic variation leading to functional differences observed across *T. brucei*, such as the SRA gene, which is specific to *Tbr* and responsible for differences in life history and clinical disease types (Gibson 2005). However, this would require multiple de novo assemblies, which for the short read data generated in this study is likely to be highly fragmented and thus of limited utility.

Admixture analysis (Alexander et al. 2009) (fig. 2) also revealed significant admixture between clusters with several individuals sharing substantial ancestry from more than one cluster. Of note is the mixed assignment of the *Te* sample EKC80 from Kenya, which shares ancestry with clusters 1, 2, 3, and 9. This confirms previous findings (Lai et al. 2008) that suggest that *Te* is similar to the three taxa within the *T. brucei* complex (*Tbb*, *Tbr,* and *Tbg*) and implies potential gene flow between *Te* and all three *T. brucei* named subspecies, although only this strain out of the four *Te* strains included in this study shows sign of admixture. The substantial gene flow observed between *T. brucei* strains further supports the lack of an evolutionary basis to the subspecies designations within *T. brucei*.

The presence of significant partitioning of genomic variation in the *T. brucei* complex due to geographic influences and host specificity should be explored using finer scale individual sampling at a regional/local scale. This finding can be explored to develop an SNP-based assay for HAT monitoring, as they provide means to identify the evolutionary and geographic origin of strains and their association with specific hosts. This is especially valuable to assess the identity of strains in the new HAT foci in central Uganda and to monitor the feared merger of the two human parasites (*Tbr* and *Tbg*) in Northern Uganda.

### Genomic Patterns of Selection and LD

Analysis of selection across the *T. brucei* genome showed that much of it is under purifying selection. This is concordant with the finding of high ratio of coding to noncoding sequence typical of the *T. brucei* genome (Berriman et al. 2005). To further investigate the targets of these selective processes, which may be driving diversity in *Tbb/Tbr/Tbg2*, we examined annotated coding regions in windows under strong diversifying selection (supplementary appendix S2, Supplementary Material online).

The finding of significant enrichment for genes associated with phosphorous–oxygen lyase function, including adenylate cyclase (table 3), is of particular interest, as it has been shown that reducing adenylate cyclase function reduces the ability of *T. brucei* to control the early innate immune defense of the host (Salmon et al. 2012). Thus, the finding of diversity in the genes associated with this function is of particular interest as it could be exploited for developing novel HAT treatment methods. Regions under high diversifying selection included two genes encoding proteins associated with the flagellum, such as a flagellum-adhesion glycoprotein and the paraflagellar rod component (supplementary appendix S2, Supplementary Material online). This is an unexpected result, as it would be generally expected that these genes would be conserved across the group, given the critical role that the flagellum plays in both the life history and pathogenicity of trypanosomatids (Ralston and Hill 2006). The fact that they are under diversifying selection suggests that variation in these genes may contribute to disease and life history variation in African trypanosomes. (Ralston et al. 2009).

Although as expected the majority of genes of known function under purifying selection was common across eukaryotes, the finding of significant enrichment for genes associated with cytoskeleton structure (table 3) suggests that dependence on a diverse range of microtubule functions is a trait common across *Tbb/Tbr/Tbg2* (Gull 1999; Gluenz et al. 2011). Although we did not detect significant enrichment for genes associated with VSG expression, many of these were present in genomic windows under high purifying selection (PAG3, ESAG3, GRESAG2, GRESAG4), indicating that conservation of these genes may be important in preserving their function. This also confirms previous suggestions that the high levels of recombination characteristic of VSG genes are not present in ESAG genes (McCulloch and Horn 2009). Given their apparent conservation in African trypanosomes, further elucidation of their functional role will provide significant insights into the mechanisms underlying antigenic variation across the group (Pays et al. 2004; Salmon et al. 2012).

LD analyses across *Tbb/Tbr/Tbg2* showed that chromosome length is positively correlated with *r*^2^. This is in line with the theory that meiotic reciprocal recombination, a common process across eukaryotes, predicts that smaller chromosomes will have higher recombination rates than larger chromosomes, resulting in a positive correlation between LD and chromosome length (Kaback et al. 1999; Jensen-Seaman et al. 2004). We found that our result was not explained by either the number of repeat annotations or the number of annotated VSG genes, despite subtelomeric VSG arrays being some of the most dynamic elements of the megabase chromosomes (Jackson et al. 2010).

## Conclusions

Comparative evaluation of *T. brucei* genomes yields several insights into the genomic arrangement and variation among *T. brucei* isolates from a broad sample (supplementary appendix S1, Supplementary Material online and fig. 2). Understanding this variation offers the ability to develop tools to monitor the future success of disease control implementations—especially the differential effects of various control measures on strains with differing disease outcomes and genomic backgrounds. In the event of re-emergence of HAT following elimination, or the feared merger of the two human disease forms in Northern Uganda, this framework offers the ability to develop a suite of genetic tools for the rapid identification of emergent and possibly co-occurring strains. This can provide crucial information to formulate appropriate responses, and represents a significant advance toward the stated goal of HAT elimination by 2020 (WHO 2012).

## Supplementary Material

Supplementary methods, appendices S1–S3, and figures S1–S3 are available at *Genome Biology and Evolution* online (http://www.gbe.oxfordjournals.org/).

Supplementary Data
