# Supplementary material for: Comparative Genomics Reveals Multiple Genetic Backgrounds of Human Pathogenicity in the Trypanosoma brucei Complex
Source: Genome Biol Evol. 2014 Oct 5;6(10):2811–9. doi: 10.1093/gbe/evu222 (PMC4224348; doi:10.1093/gbe/evu222)
Supplement: Supplementary Data [file supp_6_10_2811__index.html]

Comparative genomics reveals multiple genetic backgrounds of human pathogenicity in the Trypanosoma brucei complex — Comparative Genomics Reveals Multiple Genetic Backgrounds of Human Pathogenicity in the Trypanosoma brucei Complex — Supplementary Data 

# Comparative Genomics Reveals Multiple Genetic Backgrounds of Human Pathogenicity in the *Trypanosoma brucei* Complex

## Supplementary Data

files

**Files in this Data Supplement:**

- Supplementary Data - zip file
