# Supplementary material for: Comparative Genomics Reveals Multiple Genetic Backgrounds of Human Pathogenicity in the Trypanosoma brucei Complex
Source: Genome Biol Evol. 2014 Oct 5;6(10):2811–9. doi: 10.1093/gbe/evu222 (PMC4224348; doi:10.1093/gbe/evu222)
Supplement: Supplementary Data [file supp_evu222_suppl_data.zip › Supplementarymaterial.pdf]

Sample Name	Isolate	Subspecies	Country	Host
BIP70	TSW 187/78E	*T. brucei brucei*	Ivory Coast	Pig
BIP82	TSW 55	*T. brucei brucei*	Ivory Coast	Pig
BIP85	PTAG 129	*T. brucei brucei*	Ivory Coast	Pig
BIT89	KP33 clone 16	*T. brucei brucei*	Ivory Coast	Tsetse
BNP62	B8/18 clone B	*T. brucei brucei*	Nigeria	Pig
BDP87	SW3/87	*T. brucei brucei*	D.R. Congo	Pig
BIP92	LM 56 clone 6	*T. brucei brucei*	Ivory Coast	Pig
G2IH78	TH126	*T. brucei gambiense* group 2	Ivory Coast	Human
G1DH70	1829 (Aljo)	*T. brucei gambiense* group 1	D.R. Congo	Human
G1CH88	Fontem strain 10	*T. brucei gambiense* group 1	Cameroon	Human
EKC80	KETRI 2479	*T. evansi*	Kenya	Camel
EAU85	STIB810	*T. evansi*	China	Buffalo
EKC81	C13	*T. evansi*	Kenya	Camel
EBR85	E110	*T. evansi*	Brazil	Capybara
BUT71	STIB776	*T. brucei brucei*	Uganda	Tsetse
BKW71	KETRI1902	*T. brucei brucei*	Kenya	Waterbuck
BKT80	LF1	*T. brucei brucei*	Kenya	Tsetse
BTY71	STIB213	*T. brucei brucei*	Tanzania	Hyena
BUD05	H883	*T. brucei brucei*	Uganda	Canine
BUB03	H884	*T. brucei brucei*	Uganda	Bovine
RUH03	H880	*T. brucei rhodesiense*	Uganda	human
RUH101	H885	*T. brucei rhodesiense*	Uganda	human
RUH102	H886	*T. brucei rhodesiense*	Uganda	human
BKB80	LVBG3N	*T. brucei brucei*	Kenya	Bovine
RKH78	LVH56	*T. brucei rhodesiense*	Kenya	Human
RUH902	H866	*T. brucei rhodesiense*	Uganda	human
RUH903	H870	*T. brucei rhodesiense*	Uganda	human
RUH92	H887	*T. brucei rhodesiense*	Uganda	human
BTK71	STIB348	*T. brucei brucei*	Tanzania	Kongoni
RZH82	058	*T. brucei rhodesiense*	Zambia	Human
RTY71	STIB324	*T. brucei rhodesiense*	Tanzania	Hyena
RTH82	STIB704BA	*T. brucei rhodesiense*	Tanzania	human
BZY73	J10	*T. brucei brucei*	Zambia	Hyena
BKS70	KETRI1738	*T. brucei brucei*	Kenya	Sheep
RUH61	EATRO-0240	*T. brucei rhodesiense*	Uganda	Human
REH67	STIB809	*T. brucei rhodesiense*	Ethiopia	Human
BUS60	427 var 3	*T. brucei brucei*	Uganda	Sheep
BUB09	H879	*T. brucei brucei*	Uganda	Bovine
RUH901	H865	*T. brucei rhodesiense*	Uganda	Human

Date collected	Cluster	No. reads	Coverage (x)	Percent of reads mapped
1978	1	29,049,798	43	59.7%
1982	1	36,916,562	64	70.1%
1985	1	48,018,620	88	73.9%
1989	1	40,137,980	72	72.5%
1962	1	50,018,208	86	69.6%
1987	1	51,292,480	85	66.7%
1992	1	48,195,936	86	72.1%
1978	1	102,413,178	176	69.4%
1970	2	97,106,810	173	71.6%
1988	2	95,530,706	179	75.4%
1980	3	39,535,358	73	74.8%
1985	3	29,413,952	58	79.1%
1981	3	40,475,730	78	78.0%
1985	3	44,386,900	90	81.4%
1971	4	126,778,218	234	74.4%
1971	4	107,295,764	199	74.7%
1980	4	90,962,678	163	72.3%
1971	4	116,407,110	217	75.1%
2005	5	78,940,960	144	73.6%
2003	5	89,574,410	162	72.8%
2003	5	77,381,242	141	73.6%
2010	5	87,145,816	151	69.7%
2010	5	92,042,242	164	71.7%
1980	6	111,930,244	193	69.6%
1978	6	99,418,374	176	71.3%
1990	6	91,782,024	162	71.3%
1990	6	74,365,890	135	73.2%
1992	6	81,110,736	147	73.1%
1971	7	54,974,332	98	72.1%
1974	7	91,669,794	154	67.9%
1971	7	149,581,682	279	75.1%
1982	7	39,679,496	73	73.8%
1973	8	95,444,312	179	75.5%
1970	8	94,391,900	180	76.9%
1961	8	113,598,792	239	84.7%
1967	9	69,654,164	127	73.4%
1960	9	28,838,940	51	70.8%
2009	9	87,731,686	158	72.7%
1990	9	86,543,962	154	71.9%

GeneID	Type of annotation	No. SNPs	Tajima's D	Direction	MK Alpha
Tb927.1.1000	CDS	8	0.246312	D	
Tb927.1.1030	CDS	20	1.55601	D	
Tb927.1.1560	CDS	24	0.601157	D	
Tb927.1.2340	CDS	0	N/A	P	
Tb927.1.2350	CDS	0	N/A	P	
Tb927.1.2360	CDS	0	N/A	P	
Tb927.1.2370	CDS	0	N/A	P	
Tb927.1.2380	CDS	0	N/A	P	
Tb927.1.870	CDS	7	0.746473	D	
Tb927.10.12310	CDS	26	0.381778	D	
Tb927.10.12330	CDS	1	0	D	
Tb927.10.13160	CDS	13	-0.226017	D	
Tb927.10.13190	CDS	18	1.18513	D	
Tb927.10.1380	CDS	54	2.45506	D	
Tb927.10.1390	CDS	5	-0.387414	D	
Tb927.10.1400	CDS	4	0.58193	D	
Tb927.10.1420	CDS	13	2.39391	D	
Tb927.10.16340	CDS	6	-0.736098	P	
Tb927.10.2410	CDS	23	1.3755	D	
Tb927.10.2430	CDS	0	N/A	D	
Tb927.10.2440	CDS	33	0.881366	D	
Tb927.10.2490	CDS	20	0.0963001	D	
Tb927.10.2540	CDS	8	0.531326	D	
Tb927.10.2550	CDS	27	1.84389	D	
Tb927.10.2560	CDS	53	3.0459	D	
Tb927.10.2570	CDS	26	0.746627	D	
Tb927.10.2880	CDS	76	0.7459	D	
Tb927.10.2960	CDS	34	2.09817	D	
Tb927.10.2980	CDS	0	N/A	D	19S prote
Tb927.10.4370	CDS	0	N/A	P	expre
Tb927.10.6190	CDS	16	0.683944	D	
Tb927.10.6880	CDS	9	1.10056	D	
Tb927.10.6910	CDS	1	0	D	
Tb927.10.8900	CDS	14	1.99995	D	
Tb927.10.8920	CDS	17	2.17769	D	
Tb927.10.8930	CDS	24	0.0331948	D	0.528942
Tb927.10.8950	CDS	3	1.15758	D	
Tb927.10.8970	CDS	1	0	D	
Tb927.11.10000	CDS	4	-0.159986	D	
Tb927.11.10010	CDS	9	0.857909	D	
Tb927.11.10020	CDS	10	1.48789	D	
Tb927.11.10030	CDS	2	0.542348	D	
Tb927.11.10900	CDS	16	2.76066	D	
Tb927.11.10910	CDS	3	-1.09381	D	
Tb927.11.10930	CDS	15	1.77044	D	
Tb927.11.11980	CDS	3	-1.09033	D	

Tb927.11.12750	CDS	5	2.39726	D	NS1 effec
Tb927.11.12770	CDS	57	1.94245	D	
Tb927.11.12780	CDS	0	N/A	D	
Tb927.11.12790	CDS	0	N/A	D	
Tb927.11.12800	CDS	0	N/A	D	
Tb927.11.12810	CDS	0	N/A	D	
Tb927.11.12820	CDS	0	N/A	D	
Tb927.11.17040	CDS	0	N/A	P	denylate cy
Tb927.11.1780	CDS	19	0.667117	D	
Tb927.11.1790	CDS	6	-1.52785	D	
Tb927.11.1800	CDS	0	N/A	D	
Tb927.11.18160	CDS	0	N/A	P	expres
Tb927.11.18180	CDS	0	N/A	P	expres
Tb927.11.18180	CDS	0	N/A	P	expres
Tb927.11.18180	CDS	0	N/A	P	expres
Tb927.11.18180	CDS	0	N/A	P	expres
Tb927.11.1860	CDS	0	N/A	D	
Tb927.11.1870	CDS	0	N/A	D	
Tb927.11.1880	CDS	11	-0.011653	D	
Tb927.11.1900	CDS	11	3.05815	D	
Tb927.11.1910	CDS	16	1.85528	D	
Tb927.11.230	CDS	37	1.36021	D	
Tb927.11.250	CDS	14	1.10254	D	
Tb927.11.3120	CDS	17	1.70597	D	
Tb927.11.5220	CDS	28	0.683618	D	
Tb927.11.7420	CDS	11	2.45392	D	
Tb927.11.7450	CDS	32	0.251335	D	
Tb927.11.9270	CDS	26	1.41249	D	
Tb927.11.9310	CDS	27	2.77075	D	
Tb927.11.9330	CDS	46	2.12842	D	
Tb927.11.9350	CDS	21	2.46305	D	
Tb927.11.9360	CDS	10	1.02897	D	
Tb927.11.9990	CDS	11	0.57703	D	
Tb927.2.1310	CDS	77	0.613634	D	
Tb927.2.1330	CDS	56	2.08387	D	retrotr
Tb927.2.1344	CDS	15	2.05344	D	retrotr
Tb927.2.1350	CDS	0	N/A	D	retrotr
Tb927.2.1680	CDS	0	N/A	D	
Tb927.2.5030	CDS	13	0.846363	D	
Tb927.2.5060	CDS	6	1.50217	D	
Tb927.3.2040	CDS	23	-0.300348	P	
Tb927.3.3447	rRNA	0	N/A	D	
Tb927.4.3620	CDS	7	1.415	D	
Tb927.4.3630	CDS	1	0	D	
Tb927.4.3640	CDS	1	0	D	
Tb927.4.3880	CDS	0	N/A	D	
Tb927.4.3890	CDS	19	0.0791453	D	

Tb927.4.4870	CDS	17	-0.100949	D	
Tb927.5.2880	CDS	5	-1.48627	P	
Tb927.5.320	CDS	0	N/A	D	
Tb927.5.330	CDS	0	N/A	D	ATP
Tb927.5.3400	CDS	16	0.0648432	P	
Tb927.5.3430	CDS	23	-0.759337	P	
Tb927.5.3800	CDS	67	2.97613	D	
Tb927.5.3810	CDS	12	1.99246	D	OMF
Tb927.5.3820	CDS	17	0.785111	D	-0.15909
Tb927.5.3910	CDS	10	1.72939	D	
Tb927.5.3920	CDS	43	3.07574	D	
Tb927.5.3940	CDS	15	-0.034792	D	-0.11182
Tb927.5.4330	CDS	23	-1.15183	P	
Tb927.5.4350	CDS	2	-1.06309	P	
Tb927.5.4430	CDS	1	0	P	
Tb927.5.4440	CDS	0	N/A	P	
Tb927.5.4760	CDS	0	N/A	P	expres
Tb927.6.182	rRNA	0	N/A	P	
Tb927.6.183	rRNA	0	N/A	P	
Tb927.6.184	rRNA	0	N/A	P	
Tb927.6.185	rRNA	0	N/A	P	
Tb927.6.186	rRNA	0	N/A	P	
Tb927.6.187	rRNA	0	N/A	P	
Tb927.6.210	CDS	40	1.68917	D	le
Tb927.6.270	CDS	0	N/A	P	receptor
Tb927.6.280	CDS	0	N/A	P	
Tb927.6.290	CDS	0	N/A	P	receptor
Tb927.6.300	CDS	0	N/A	P	
Tb927.6.390	CDS	3	-0.550131	D	
Tb927.6.460	CDS	0	N/A	P	
Tb927.6.470	CDS	0	N/A	P	
Tb927.6.480	CDS	0	N/A	P	form specif
Tb927.6.490	CDS	0	N/A	P	
Tb927.6.4910	CDS	0	N/A	D	
Tb927.6.4920	CDS	0	N/A	D	
Tb927.6.4950	CDS	3	0.321151	D	
Tb927.6.4960	CDS	15	1.45187	D	
Tb927.6.4970	CDS	28	1.90315	D	
Tb927.6.940	CDS	6	1.42235	D	
Tb927.6.950	CDS	24	-0.19232	D	
Tb927.6.960	CDS	0	N/A	D	
Tb927.6.970	CDS	0	N/A	D	
Tb927.6.980	CDS	0	N/A	D	
Tb927.7.170	CDS	0	N/A	D	expre
Tb927.7.190	CDS	56	1.62851	D	
Tb927.7.210	CDS	25	1.81547	D	
Tb927.7.220	CDS	8	2.49688	D	

Tb927.7.230	CDS	1	0	D	
Tb927.7.240	CDS	2	1.38672	D	
Tb927.7.250	CDS	22	1.38269	D	
Tb927.7.270	CDS	22	1.82849	D	
Tb927.7.280	CDS	9	1.73018	D	
Tb927.7.540	CDS	15	2.32022	D	
Tb927.7.5580	CDS	135	-0.984783	D	
Tb927.7.570	CDS	5	2.03672	D	
Tb927.7.580	CDS	24	0.418101	D	
Tb927.7.6050	CDS	0	N/A	P	
Tb927.7.6060	CDS	0	N/A	P	
Tb927.7.920	CDS	58	2.58148	D	
Tb927.7.930	CDS	8	1.10918	D	
Tb927.7.940	CDS	10	2.79753	D	
Tb927.8.1310	CDS	9	1.16955	D	enine nucle
Tb927.8.1330	CDS	1	0	D	
Tb927.8.1340	CDS	1	0	D	
Tb927.8.200	CDS	35	-1.11137	P	
Tb927.8.2160	CDS	85	1.68414	D	
Tb927.8.2200	CDS	8	0.749432	D	
Tb927.8.2210	CDS	19	0.876496	D	
Tb927.8.230	CDS	0	N/A	P	expres
Tb927.8.2310	CDS	5	0.91448	D	
Tb927.8.3220	CDS	28	0.451715	D	
Tb927.8.3690	CDS	18	1.15513	D	
Tb927.8.4090	CDS	0	N/A	D	
Tb927.8.4110	CDS	12	1.93327	D	
Tb927.8.4120	CDS	0	N/A	D	
Tb927.8.4130	CDS	15	1.37916	D	
Tb927.8.4170	CDS	41	0.831781	D	
Tb927.8.6840	CDS	25	0.462713	D	
Tb927.8.6880	CDS	25	0.453988	D	
Tb927.8.6910	CDS	4	2.25124	D	
Tb927.8.6930	CDS	40	0.0796877	D	
Tb927.8.7620	CDS	0	N/A	P	
Tb927.8.7630	CDS	1	0	P	
Tb927.8.7640	CDS	12	-1.38394	P	
Tb927.8.7650	CDS	16	-1.01289	P	
Tb927.8.7860	CDS	0	N/A	P	
Tb927.8.7870	CDS	0	N/A	P	
Tb927.8.7880	CDS	0	N/A	P	recepto
Tb927.8.7890	CDS	0	N/A	P	
Tb927.9.14400	CDS	8	-1.2335	P	
Tb927.9.14410	CDS	6	-0.683243	P	
Tb927.9.14420	CDS	7	0.476175	P	
Tb927.9.14430	CDS	4	-1.07013	P	
Tb927.9.14470	CDS	30	-1.27478	P	

Tb927.9.3460	CDS	43	1.87171	D	
Tb927.9.3470	CDS	8	0.373178	D	
Tb927.9.3480	CDS	3	0.0214299	D	
Tb927.9.3530	CDS	6	2.27127	D	
Tb927.9.3990	CDS	3	-0.997971	P	
Tb927.9.4040	CDS	0	N/A	P	
Tb927.9.450	CDS	0	N/A	D	[illegible]
Tb927.9.450	CDS	0	N/A	D	[illegible]
Tb927.9.450	CDS	0	N/A	D	[illegible]
Tb927.9.4620	CDS	14	0.226741	D	
Tb927.9.480	CDS	0	N/A	D	[illegible]
Tb927.9.560	CDS	0	N/A	P	[illegible]
Tb927.5.295	CDS			D	
27_08_v4.snoRNA.[illegible]	ncRNA			D	
27_08_v4.snoRNA.[illegible]	ncRNA			D	
27_08_v4.snoRNA.[illegible]	ncRNA			D	
27_08_v4.snoRNA.[illegible]	ncRNA			D	
27_08_v4.snoRNA.[illegible]	ncRNA			D	
27_08_v4.snoRNA.[illegible]	ncRNA			D	
Tb09_rRNA_4	rRNA			P	
Tb09_rRNA_5	rRNA			P	
Tb09_rRNA_6	rRNA			P	

**Gene name**
developmentally regulated phosphoprotein
leucine-rich repeat protein (LRRP), putative
N-ethylmaleimide sensitive factor (NsF) vesicular-fusion protein nsf, putative
alpha tubulin
beta tubulin
alpha tubulin
beta tubulin
alpha tubulin
deoxyhypusine synthase, putative
helicase-like protein
zinc finger protein family member, putative
mannosyltransferase-II, putative
ARP2/3 complex subunit, putative actin related protein 2/3 complex, putative
serine/threonine-protein kinase, putative
hypoxanthine-guanine phosphoribosyltransferase,putative
hypoxanthine-guanine phosphoribosyltransferase
metallo- peptidase, Clan MG, Family M24 methionine aminopeptidase 2, putative
UDP-Gal or UDP-GlcNAc-dependentglycosyltransferase (pseudogene), putative
MSP-C, putative
receptor-type adenylate cyclase GRESAG 4,putative
cysteine peptidase, Clan CD, family C13,putative metacaspase MCA4
glucose-6-phosphate 1-dehydrogenase
adenylate kinase, putative
malate dehydrogenase-related
mitochondrial malate dehydrogenase
lysosomal alpha-mannosidase precursor, putative
calcium channel protein, putative
elongation factor, putative GTP-binding protein, putative
asome regulatory subunit,Metallo-peptidase, Clan MP, Family M67 proteasome regulatory non-ATPase s
ssion site-associated gene 3 (ESAG3)protein, putative expression site-associated gene (ESAG) protein,pu
aldehyde dehydrogenase, putative
glyceraldehyde 3-phosphate dehydrogenase,cytosolic
Sterol methyltransferase, putative
choline/ethanolamine phosphotransferase (CEPT)
ras-like small GTPase, putative
paraflagellar rod component, putative
kinetoplast DNA-associated protein, putative
kinetoplast DNA-associated protein, putative
SNF7-like protein, putative class-E vacuolar protein-sorting protein 24(Vps24p), putative
vacuolar sorting-associated protein-like,putative
short-chain dehydrogenase, putative
60S ribosomal protein L29, putative
MENG
40S ribosomal protein SA, putative
tubulin delta chain delta tubulin
corset-associated protein 15 microtubule-associated protein

:tor domain- binding protein 1 CPSF 30 kDa subunit cleavage and polyadenylation specificity factor30 kD
ubiquitin ligase, putative
ribonucleoside-diphosphate reductase smallchain, putative
ribonucleoside-diphosphate reductase smallchain
ribonucleoside-diphosphate reductase smallchain
ribonucleoside-diphosphate reductase smallchain
ribonucleoside-diphosphate reductase smallchain
/clase, putative expression site-associated gene 4 (ESAG4)protein, putative expression site-associated ge
protein phosphatase 2C, putative
histone H1, putative
histone H1, putative
ssion site-associated gene 3 (ESAG3),degenerate expression site-associated gene (ESAG,pseudogene), pu
ssion site-associated gene 3 (ESAG3),degenerate expression site-associated gene (ESAG,pseudogene), pu
ssion site-associated gene 3 (ESAG3),degenerate expression site-associated gene (ESAG,pseudogene), pu
ssion site-associated gene 3 (ESAG3),degenerate expression site-associated gene (ESAG,pseudogene), pu
ssion site-associated gene 3 (ESAG3),degenerate expression site-associated gene (ESAG,pseudogene), pu
histone H1, putative
histone H1, putative
histone H1, putative
T-complex protein 1, beta subunit, putative
cation transporter protein, putative
cleavage and polyadenylation specificity factor,putative
cysteine peptidase, Clan CA, family C12,putative ubiquitin carboxyl-terminal hydrolase, putative
nucleolar GTP-binding protein 1
chaperone protein DNAj, putative
phosphoadenosine phosphosulfate reductase-likeprotein
zinc finger protein family member, putative
zinc finger protein kinase protein kinase
tRNA pseudouridine synthase, putative
helicase-like protein
phosphonopyruvate decarboxylase-like protein,putative
prenyltransferase, putative
glycosyl transferase-like protein
leucine-rich repeat protein (LRRP), frameshift leucine-rich repeat protein (LRRP, pseudogene),putative
ansposon hot spot protein 6 (RHS6),degenerate retrotransposon hot spot protein (RHS,pseudogene), pu
ansposon hot spot protein 1 (RHS1),interrupted retrotransposon hot spot protein (RHS,pseudogene), pu
ansposon hot spot protein 1 (RHS1),interrupted retrotransposon hot spot protein (RHS,pseudogene), pu
cyclophilin-type peptidyl-prolyl cis-transisomerase, putative
transcription initiation protein, putative
GTP binding protein, putative
kinesin, putative
rRNA small subunit rRNA
protein phosphatase 1, putative
protein phosphatase 1, putative
protein phosphatase 1, putative
receptor-type adenylate cyclase GRESAG 4,putative
ATP-dependent RNA helicase, putative

amino acid transporter, putative
chaperone protein DNAj, putative
adenylyl cyclase, putative receptor-type adenylate cyclase GRESAG 4,putative
pyrophosphate-lyase, putative adenylyl cyclase, putative receptor-type adenylate cyclase GRESAG 4,puta
calcium pump calcium-translocating P-type ATPase
ubiquitin-activating enzyme e1, putative
glutamine hydrolysing (not ammonia-dependent)carbomoyl phosphate synthase, putative
'DCase-OPRTase, putative orotidine-5-phosphate decarboxylase/orotatephosphoribosyltransferase, puta
aspartate carbamoyltransferase, putative
small nuclear RNA-activating protein
peroxisome assembly protein, putative
helicase, putative
dihydrolipoamide branched chain transacylase,putative
NUDIX hydrolase, putative
protein kinase, putative
dynein light chain, putative
ssion site-associated gene 3 (ESAG3),degenerate expression site-associated gene (ESAG,pseudogene), pu
rRNA large subunit zeta (M6)
rRNA large subunit delta (M2)
rRNA large subunit beta rRNA
rRNA large subunit beta
rRNA large subunit gamma (M1)
rRNA large subunit alpha
eucine-rich repeat protein 1 (LRRP1),degenerate leucine-rich repeat protein (LRRP, pseudogene),putative
r-type adenylate cyclase GRESAG 4,degenerate receptor-type adenylate cyclase GRESAG 4,pseudogene,
receptor-type adenylate cyclase GRESAG 4,putative
r-type adenylate cyclase GRESAG 4,degenerate receptor-type adenylate cyclase GRESAG 4,pseudogene,
receptor-type adenylate cyclase GRESAG 4,putative
adenylyl cyclase, degenerate adenylyl cyclase, pseudogene, putative
procyclic acidic repetitive protein A procyclin PARP A procyclin associated gene 3 (PAG3) protein
gene related to expression site-associated gene2 (GRESAG2), putative
'ic polypeptide A-betaprecursor surface protein EP3-2 procyclin precursor surface protein EP3-2 PARP A-
procyclic acidic repetitive protein A procyclin PARP A procyclin associated gene 3 (PAG3) protein
S-adenosylmethionine synthetase, putative
S-adenosylmethionine synthetase, putative
mago nashi-like protein, putative
zinc finger-domain protein, putative
protein kinase, putative serine/arginine-rich protein specific kinaseSRPK, putative
metacaspase MCA2
cysteinyl-tRNA synthetase, putative
cysteine peptidase, Clan CA, family C1,Cathepsin L-like cysteine peptidase precursor
cysteine peptidase, Clan CA, family C1,Cathepsin L-like cysteine peptidase precursor
cysteine peptidase, Clan CA, family C1,Cathepsin L-like cysteine peptidase precursor
ssion site-associated gene 9 (ESAG9)protein, putative expression site-associated gene (ESAG) protein,pu
metallo- peptidase, Clan MA(E) Family M3,putative thimet oligopeptidase A, putative
proline dehydrogenase
CDP-diacylglycerol synthetase, putative

40S ribosomal protein S33, putative
40S ribosomal protein S33, putative
zinc finger protein family member, putative
ribosome biogenesis protein, putative
cyclophilin-type peptidyl-prolyl cis-transisomerase, putative
chaperone protein DNAj, putative
cell cycle associated protein MOB1-B
prefoldin, putative
GTP-binding protein, putative
receptor-type adenylate cyclase GRESAG 4,putative
receptor-type adenylate cyclase GRESAG 4,putative
dynein heavy chain, putative
zinc finger protein family member, putative
glucosidase II beta subunit, putative protein kinase C substrate protein, heavy chain,putative
eotide mitochondrial translocator,putative ADP/ATP mitochondrial translocase, putative mitochondrial c
60S ribosomal protein L7a, putative
60S ribosomal protein L7a, putative
UDP-Gal or UDP-GlcNAc-dependentglycosyltransferase (pseudogene), putative
p-glycoprotein multidrug resistance protein A
terbinafine resistance locus protein (yip1),putative
pteridine reductase
sion site-associated gene 3 (ESAG3),degenerate expression site-associated gene (ESAG,pseudogene), pu
(H+)-ATPase G subunit, putative
exonuclease, putative
isocitrate dehydrogenase [NADP], mitochondrialprecursor, putative
endonuclease G, putative
flagellum-adhesion glycoprotein, putative
zinc finger protein family member, putative
class I transcription factor A, subunit 5b
RNA-binding protein, putative
mismatch repair protein MLH1
translation initiation factor IF-2, putative
cyclophilin, putative
serine/threonine-protein kinase NrkA
amino acid transporter 1, putative
amino acid transporter 1, putative
amino acid transporter 1, putative
amino acid transporter, putative
receptor-type adenylate cyclase GRESAG 4,putative
receptor-type adenylate cyclase GRESAG 4,putative
r-type adenylate cyclase GRESAG 4,fragment receptor-type adenylate cyclase GRESAG 4(pseudogene), p
receptor-type adenylate cyclase GRESAG 4,putative
leucine-rich repeat protein (LRRP), putative
RNA 3'-terminal phosphate cyclase-like protein
cyclophilin-like protein, putative
casein kinase II, putative
cysteine peptidase, Clan CA, family C19,putative ubiquitin carboxyl-terminal hydrolase, putative

zinc finger protein family member, putative
low molecular weight protein tyrosinephosphatase, putative
U5Cwc21 small nuclear ribonucleoprotein
Sm- like protein U6 snRNA-associated Sm-like protein LSm6p
ribosomal protein S7, putative
nicotinamidase, putative
expression site-associated gene 3 (ESAG3),fragment expression site-associated gene (ESAG,pseudogene)
expression site-associated gene 3 (ESAG3),fragment expression site-associated gene (ESAG,pseudogene)
expression site-associated gene 3 (ESAG3),fragment expression site-associated gene (ESAG,pseudogene)
ubiquitin-activating enzyme e1, putative
expression site-associated gene 3 (ESAG3),fragment expression site-associated gene (ESAG,pseudogene)
expression site-associated gene 3 (ESAG3),fragment expression site-associated gene (ESAG,pseudogene)
retrotransposon hotspot (RHS) protein 1
C/D snoRNA
C/D snoRNA
C/D snoRNA
H/ACA-like snoRNA
H/ACA-like snoRNA
H/ACA-like snoRNA
rRNA M2
rRNA M6
rRNA M4

subunit 11
tative

a subunit

ne (ESAG) protein,putative

ıtative
ıtative
ıtative
ıtative
ıtative

ıtative
ıtative
ıtative

ative

ative

utative

e
putative

putative

beta EP3-2 procyclin

tative

arrier protein

tative

utative

Gene ID	P_nonsyn	P_syn	D_nonsyn	D_syn	neutrality.index	alpha
Tb927.1.1700	0	1	346	150	0	1
Tb927.1.1720	0	1	342	56	0	1
Tb927.1.3000	0	3	184	179	0	1
Tb927.1.3110	0	1	214	53	0	1
Tb927.1.3150	0	1	330	48	0	1
Tb927.3.3690	0	2	186	259	0	1
Tb927.3.4910	0	1	337	38	0	1
Tb927.3.5100	0	1	749	91	0	1
Tb927.3.5540	0	1	287	215	0	1
Tb927.4.4380	0	1	624	30	0	1
Tb927.4.4940	0	1	534	79	0	1
Tb927.4.5050	0	1	283	231	0	1
Tb927.5.3010	0	1	55	283	0	1
Tb927.5.3660	0	1	389	156	0	1
Tb927.5.3830	0	1	89	141	0	1
Tb927.5.3970	0	1	120	117	0	1
Tb927.5.4040	0	1	302	379	0	1
Tb927.6.4990	0	1	51	85	0	1
Tb927.7.4000	0	1	405	145	0	1
Tb927.7.4460	0	2	890	131	0	1
Tb927.7.4870	0	1	261	26	0	1
Tb927.7.5140	0	1	299	153	0	1
Tb927.7.5460	0	1	173	65	0	1
Tb927.7.5590	0	1	1676	269	0	1
Tb927.7.5640	0	2	175	53	0	1
Tb927.7.5780	0	1	119	12	0	1
Tb927.8.3800	0	1	592	75	0	1
Tb927.8.4370	0	1	889	121	0	1
Tb927.8.4410	0	2	325	56	0	1
Tb927.8.5330	0	1	110	90	0	1
Tb927.8.5600	0	1	162	136	0	1
Tb927.8.5830	0	1	155	66	0	1
Tb927.8.6270	0	1	450	171	0	1
Tb927.8.7740	0	1	400	101	0	1
Tb927.8.8010	0	1	744	100	0	1
Tb927.9.6160	0	1	1045	175	0	1
Tb927.9.6510	0	1	335	228	0	1
Tb927.9.9580	0	1	557	50	0	1
Tb927.10.5380	0	2	848	272	0	1
Tb927.10.5670	0	1	432	311	0	1
Tb927.10.5980	0	1	423	56	0	1
Tb927.10.6410	0	1	820	206	0	1
Tb927.10.6800	0	2	19	204	0	1
Tb927.10.7840	0	1	238	88	0	1
Tb927.10.8820	0	2	640	169	0	1
Tb927.10.9120	0	1	64	112	0	1

Tb927.10.9300	0	1	278	65	0	1
Tb927.10.9670	0	1	598	184	0	1
Tb927.10.9820	0	1	164	337	0	1
Tb927.10.9830	0	1	77	56	0	1
Tb927.11.3710	0	1	403	143	0	1
Tb927.11.4960	0	1	152	78	0	1
Tb927.11.4970	0	1	316	174	0	1
Tb927.11.5090	0	1	226	146	0	1
Tb927.11.5190	0	1	1328	208	0	1
Tb927.11.5310	0	3	561	144	0	1
Tb927.11.5450	0	2	338	230	0	1
Tb927.11.5710	0	1	288	159	0	1
Tb927.11.5870	0	1	182	187	0	1
Tb927.11.7170	0	1	159	225	0	1
Tb927.11.7240	0	1	221	222	0	1
Tb927.11.8110	0	1	166	149	0	1
Tb927.11.8210	0	1	1008	425	0	1
Tb927.11.8860	0	1	405	266	0	1
Tb927.11.9130	0	1	170	106	0	1
Tb927.11.9180	0	2	241	84	0	1
Tb927.9.10920	0	1	640	169	0	1
Tb927.9.12040	0	1	396	111	0	1
Tb927.9.12650	0	3	550	501	0	1
Tb927.9.13010	0	1	281	182	0	1
Tb927.9.13520	0	1	729	206	0	1
Tb927.10.10070	0	1	406	93	0	1
Tb927.10.11090	0	1	555	202	0	1
Tb927.10.11950	0	1	198	32	0	1
Tb927.10.12240	0	1	158	130	0	1
Tb927.10.12480	0	1	302	41	0	1
Tb927.10.13590	0	1	850	192	0	1
Tb927.11.10170	0	1	93	96	0	1
Tb927.11.10460	0	2	186	102	0	1
Tb927.11.10860	0	1	345	79	0	1
Tb927.11.11450	0	1	625	167	0	1
Tb927.11.11460	0	1	457	87	0	1
Tb927.11.11710	0	1	313	59	0	1
Tb927.11.11850	0	1	193	416	0	1
Tb927.11.11940	0	1	62	169	0	1
Tb927.11.12220	0	1	295	382	0	1
Tb927.11.13890	0	1	118	74	0	1
Tb927.11.14090	0	1	198	194	0	1
Tb927.11.14340	0	1	606	400	0	1
Tb927.11.14550	0	1	620	104	0	1
Tb927.10.4050	1	2	497	43	0.043259557	0.95674
Tb927.10.14030	1	1	518	23	0.044401544	0.955598
Tb927.7.4210	1	1	91	5	0.054945055	0.945055

Tb927.11.11490	1	1	681	45	0.066079295	0.933921
Tb927.10.15300	1	2	1040	170	0.081730769	0.918269
Tb927.9.8350	1	1	599	53	0.088480801	0.911519
Tb927.9.10500	1	1	478	45	0.094142259	0.905858
Tb927.11.6790	1	1	560	57	0.101785714	0.898214
Tb927.11.4800	1	1	438	45	0.102739726	0.89726
Tb927.8.5710	1	2	835	199	0.119161677	0.880838
Tb927.11.8170	1	1	528	63	0.119318182	0.880682
Tb927.8.5100	1	1	2865	343	0.119720768	0.880279
Tb927.10.11730	2	2	683	93	0.136163982	0.863836
Tb927.11.16800	1	1	868	120	0.138248848	0.861751
Tb927.10.7490	1	1	721	111	0.153952843	0.846047
Tb927.9.10870	1	1	316	51	0.161392405	0.838608
Tb927.11.6400	2	1	967	80	0.165460186	0.83454
Tb927.11.7530	1	1	636	107	0.168238994	0.831761
Tb927.1.1370	1	1	518	97	0.187258687	0.812741
Tb927.11.10450	1	2	292	113	0.193493151	0.806507
Tb927.11.5020	2	1	708	73	0.206214689	0.793785
Tb927.8.7480	1	1	503	104	0.206759443	0.793241
Tb927.8.7360	3	3	864	182	0.210648148	0.789352
Tb927.10.11330	1	1	199	42	0.211055276	0.788945
Tb927.5.3850	1	1	753	159	0.211155378	0.788845
Tb927.6.3520	1	1	473	101	0.213530655	0.786469
Tb927.8.5110	1	1	1106	241	0.217902351	0.782098
Tb927.8.5290	1	1	532	122	0.229323308	0.770677
Tb927.10.13030	2	1	732	90	0.245901639	0.754098
Tb927.11.9160	1	1	390	104	0.266666667	0.733333
Tb927.11.4520	2	1	1478	203	0.274695535	0.725304
Tb927.11.8400	2	1	530	81	0.305660377	0.69434
Tb927.11.10630	2	1	883	137	0.310305776	0.689694
Tb927.10.9090	2	1	232	36	0.310344828	0.689655
Tb927.11.12110	2	2	913	298	0.326396495	0.673604
Tb927.11.10210	2	1	944	158	0.334745763	0.665254
Tb927.11.4640	1	1	642	215	0.334890966	0.665109
Tb927.8.6470	3	3	408	143	0.350490196	0.64951
Tb927.8.5240	6	2	1128	132	0.35106383	0.648936
Tb927.7.4960	2	1	457	81	0.354485777	0.645514
Tb927.10.3970	2	1	484	86	0.355371901	0.644628
Tb927.10.11270	2	1	226	41	0.362831858	0.637168
Tb927.5.3950	1	2	159	118	0.371069182	0.628931
Tb927.11.5100	2	1	821	154	0.375152253	0.624848
Tb927.7.5120	1	1	298	113	0.379194631	0.620805
Tb927.7.5200	2	1	496	96	0.387096774	0.612903
Tb927.11.8710	2	1	376	74	0.393617021	0.606383
Tb927.7.6640	2	1	1359	270	0.397350993	0.602649
Tb927.9.15400	2	1	2851	567	0.397755174	0.602245
Tb927.1.2110	1	1	509	203	0.398821218	0.601179

Tb927.6.3500	3	2	1668	455	0.409172662	0.590827
Tb927.11.11310	1	2	232	192	0.413793103	0.586207
Tb927.10.13330	3	1	1338	188	0.421524664	0.578475
Tb927.9.6110	2	1	827	175	0.423216445	0.576784
Tb927.7.5210	3	1	800	113	0.42375	0.57625
Tb927.7.5160	2	1	241	52	0.43153527	0.568465
Tb927.3.4550	4	1	621	68	0.438003221	0.561997
Tb927.11.3670	2	1	351	77	0.438746439	0.561254
Tb927.10.10690	2	1	312	71	0.455128205	0.544872
Tb927.7.4530	3	1	695	107	0.461870504	0.538129
Tb927.1.3940	2	1	787	182	0.462515883	0.537484
Tb927.5.4290	1	1	235	109	0.463829787	0.53617
Tb927.10.8930	1	1	501	236	0.471057884	0.528942
Tb927.7.4890	2	1	707	170	0.480905233	0.519095
Tb927.10.12250	4	1	801	101	0.504369538	0.49563
Tb927.8.7760	3	1	839	143	0.511323004	0.488677
Tb927.8.5630	1	1	229	119	0.519650655	0.480349
Tb927.9.12800	3	2	755	298	0.59205298	0.407947
Tb927.10.9560	2	1	272	81	0.595588235	0.404412
Tb927.11.9880	1	1	276	166	0.601449275	0.398551
Tb927.10.9140	2	1	592	187	0.631756757	0.368243
Tb927.10.11400	1	1	266	169	0.635338346	0.364662
Tb927.7.5500	3	1	1068	228	0.640449438	0.359551
Tb927.8.6420	1	1	299	196	0.655518395	0.344482
Tb927.10.7200	3	1	760	168	0.663157895	0.336842
Tb927.11.11870	2	1	270	94	0.696296296	0.303704
Tb927.8.5750	12	3	820	143	0.697560976	0.302439
Tb927.1.2580	4	1	309	54	0.699029126	0.300971
Tb927.8.5210	5	1	771	114	0.739299611	0.2607
Tb927.9.6270	1	1	159	123	0.773584906	0.226415
Tb927.10.12260	1	1	223	176	0.789237668	0.210762
Tb927.5.3900	2	1	407	168	0.825552826	0.174447
Tb927.8.4950	3	1	751	212	0.846870839	0.153129
Tb927.11.9080	1	1	548	468	0.854014599	0.145985
Tb927.10.11130	4	1	907	206	0.908489526	0.09151
Tb927.11.8010	1	1	254	232	0.913385827	0.086614
Tb927.5.4080	6	1	456	70	0.921052632	0.078947
Tb927.10.15360	7	1	1622	219	0.94512947	0.054871
Tb927.7.3910	1	1	231	222	0.961038961	0.038961
Tb927.5.3030	2	2	548	534	0.974452555	0.025547
Tb927.11.4950	1	1	335	340	1.014925373	-0.01493
Tb927.5.3610	3	2	705	482	1.025531915	-0.02553
Tb927.8.5740	2	1	100	52	1.04	-0.04
Tb927.9.7030	6	1	1721	301	1.04938989	-0.04939
Tb927.1.2990	5	4	347	307	1.105907781	-0.10591
Tb927.5.3940	2	1	313	174	1.111821086	-0.11182
Tb927.9.6670	8	1	2608	373	1.144171779	-0.14417

Tb927.5.3820	2	1	176	102	1.159090909	-0.15909
Tb927.10.5210	3	1	389	154	1.187660668	-0.18766
Tb927.11.4430	10	1	815	97	1.190184049	-0.19018
Tb927.8.5730	4	3	203	195	1.280788177	-0.28079
Tb927.9.12750	2	1	199	130	1.306532663	-0.30653
Tb927.8.3350	5	1	883	234	1.325028313	-0.32503
Tb927.10.13220	7	1	890	182	1.431460674	-0.43146
Tb927.1.3050	5	1	426	123	1.443661972	-0.44366
Tb927.8.6520	2	1	228	166	1.456140351	-0.45614
Tb927.11.9240	17	2	1738	302	1.47698504	-0.47699
Tb927.11.5490	6	1	1409	352	1.498935415	-0.49894
Tb927.11.13540	5	1	457	139	1.520787746	-0.52079
Tb927.8.5690	1	1	117	178	1.521367521	-0.52137
Tb927.10.11310	2	2	109	187	1.71559633	-0.7156
Tb927.11.4510	8	1	1754	394	1.797035348	-0.79704
Tb927.1.3030	3	2	145	204	2.110344828	-1.11034
Tb927.11.16700	3	1	566	402	2.130742049	-1.13074
Tb927.5.3580	2	1	79	95	2.405063291	-1.40506
Tb927.10.11250	2	1	319	420	2.63322884	-1.63323
Tb927.11.7270	1	1	31	99	3.193548387	-2.19355

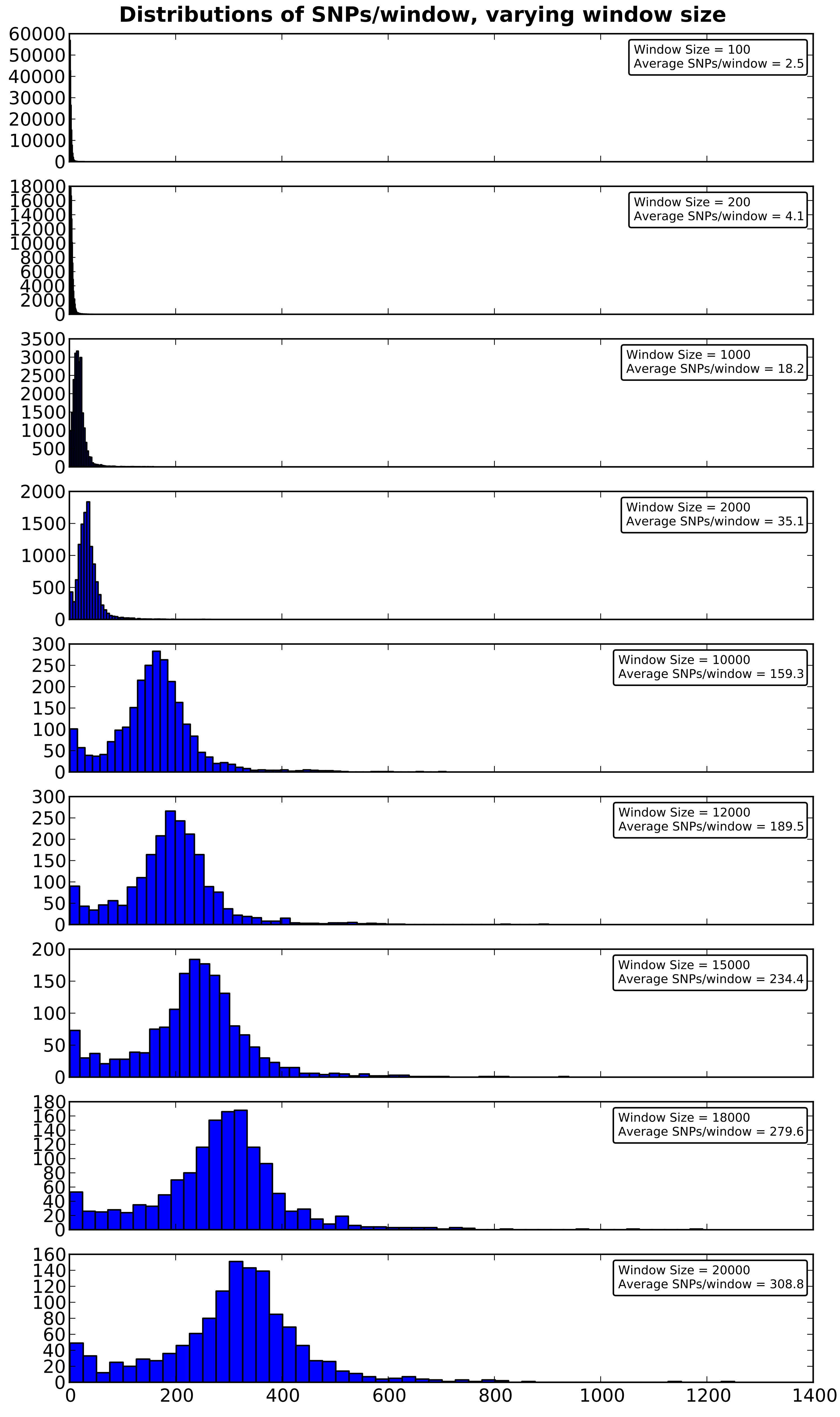
Distributions of SNPs/window, varying window size
Window Size = 100
Average SNPs/window = 2.5
Window Size = 200
Average SNPs/window = 4.1
Window Size = 1000
Average SNPs/window = 18.2
Window Size = 2000
Average SNPs/window = 35.1
Window Size = 10000
Average SNPs/window = 159.3
Window Size = 12000
Average SNPs/window = 189.5
Window Size = 15000
Average SNPs/window = 234.4
Window Size = 18000
Average SNPs/window = 279.6
Window Size = 20000
Average SNPs/window = 308.8
0
200
400
600
800
1000
1200
1400

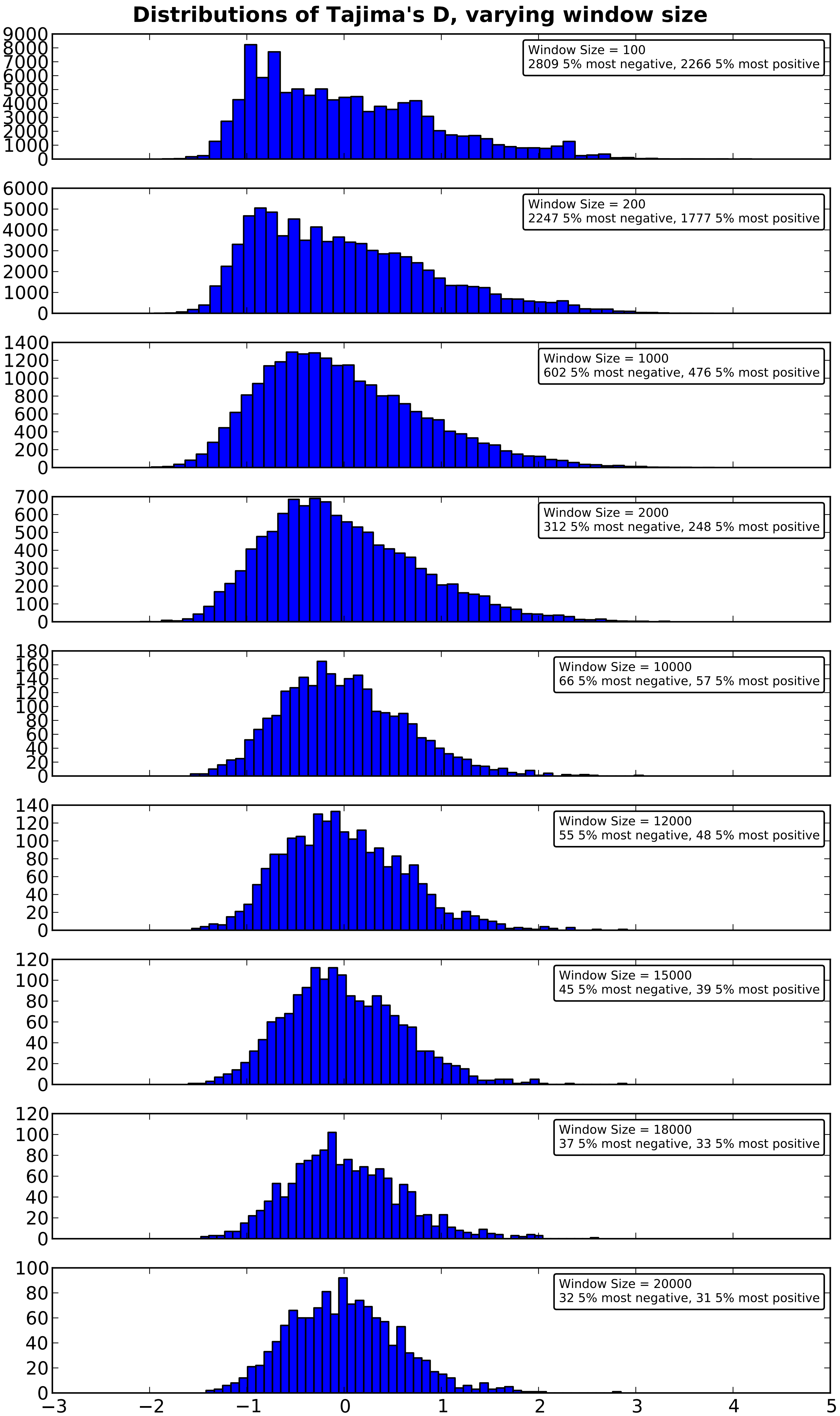

Distributions of Tajima's D, varying window size
Window Size = 100
2809 5% most negative, 2266 5% most positive
Window Size = 200
2247 5% most negative, 1777 5% most positive
Window Size = 1000
602 5% most negative, 476 5% most positive
Window Size = 2000
312 5% most negative, 248 5% most positive
Window Size = 10000
66 5% most negative, 57 5% most positive
Window Size = 12000
55 5% most negative, 48 5% most positive
Window Size = 15000
45 5% most negative, 39 5% most positive
Window Size = 18000
37 5% most negative, 33 5% most positive
Window Size = 20000
32 5% most negative, 31 5% most positive
−3
−2
−1
0
1
2
3
4
5

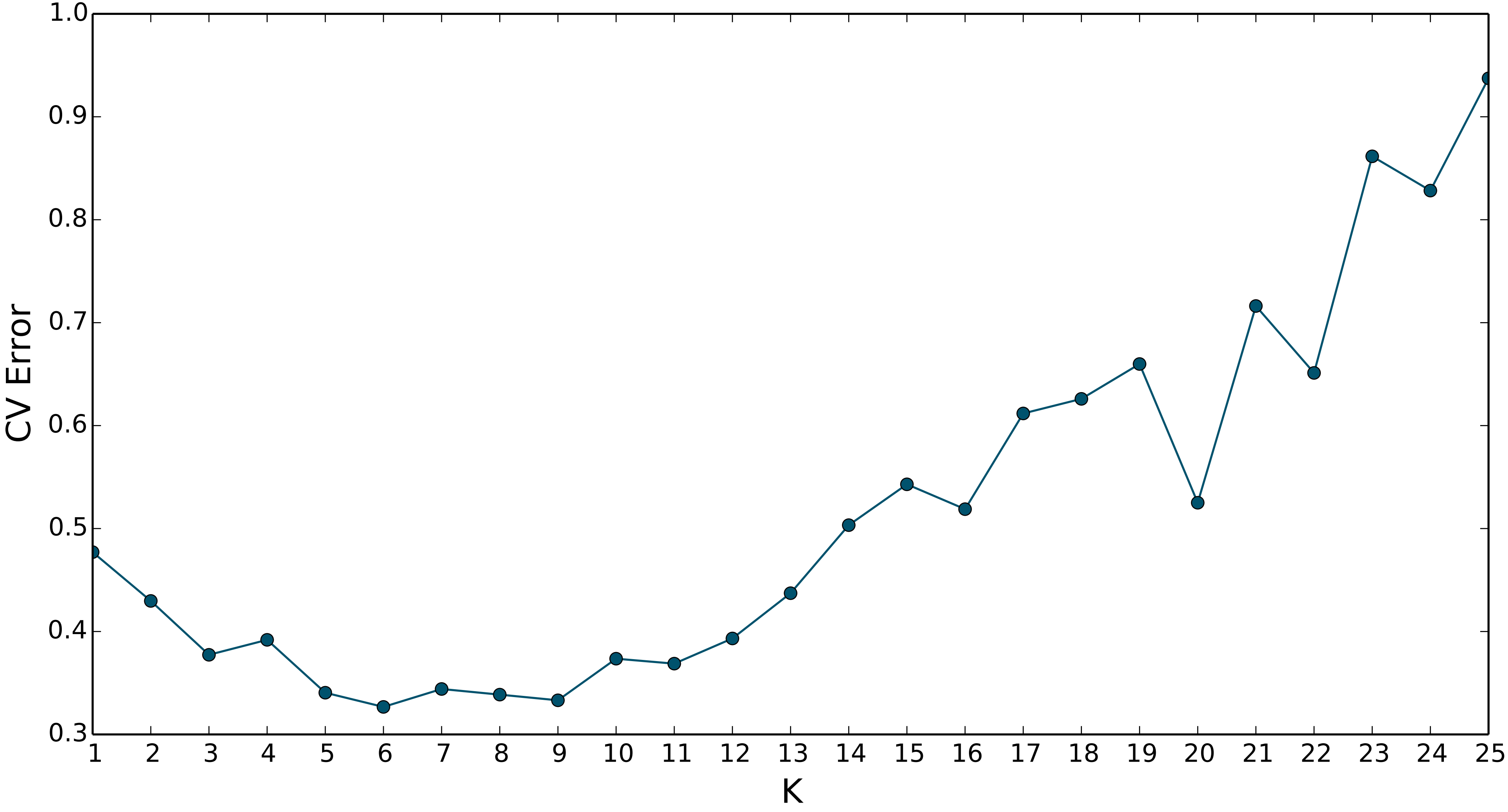

CV Error
1.0
0.9
0.8
0.7
0.6
0.5
0.4
0.3
1
2
3
4
5
6
7
8
9
10
11
12
13
14
15
16
17
18
19
20
21
22
23
24
25
K
