# Supplementary material for: Comparative Genomics Reveals Multiple Genetic Backgrounds of Human Pathogenicity in the Trypanosoma brucei Complex
Source: Genome Biol Evol. 2014 Oct 5;6(10):2811–9. doi: 10.1093/gbe/evu222 (PMC4224348; doi:10.1093/gbe/evu222)
Supplement: Supplementary Data [file supp_evu222_suppl_data.zip › Supplementarymethods.docx]

**Supplementary methods**

We used the method employed in vcftools v 1.10 to calculate Tajima’s D at the non-overlapping window sizes, 100 bp, 200 bp, 1000 bp, 2000 bp, 8000 bp, 10000 bp, 12000 bp, 15000 bp, 18000 bp, 18000 bp and 20000 bp across the megabase chromosomes (calcWINSIZE.Tajima.D, supplemental data). The distribution of the values found is summarized in Supplementary Figure 1, and the distribution of snps in each of these window sizes in Supplementary Figure 2.

We then took the windows where the highest and lowest 5% of Tajima’s D values were found for each window size and generated a BED file representing each (top_tajD_WINSIZE.bed, Supplementary data). We then used bedtools v2.17.0 to find CDS from these windows, which are summarized in gff format (top_tajD_WINSIZE_genes.gff, Supplementary data) and in gff format with the original bed file as the first four columns (top_tajD_WINSIZE_genes.txt, Supplementary data).
