# Supplementary figures and images for: Comparative Genomics Reveals Multiple Genetic Backgrounds of Human Pathogenicity in the Trypanosoma brucei Complex
Source: Genome Biol Evol. 2014 Oct 5;6(10):2811–9. doi: 10.1093/gbe/evu222 (PMC4224348; doi:10.1093/gbe/evu222)

Distributions of SNPs/window, varying window size

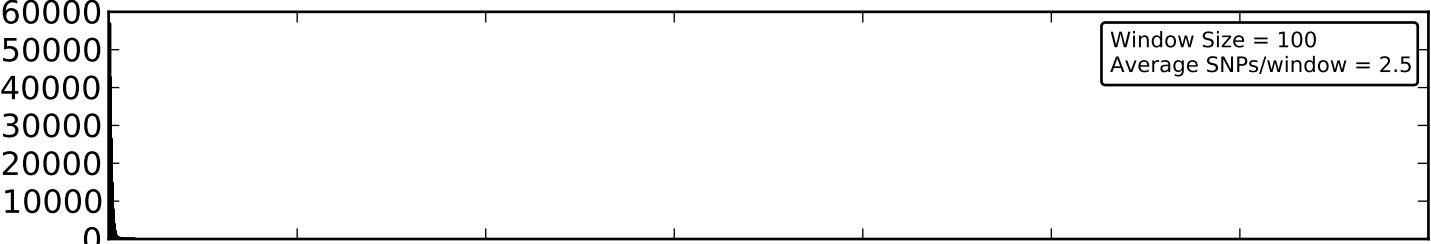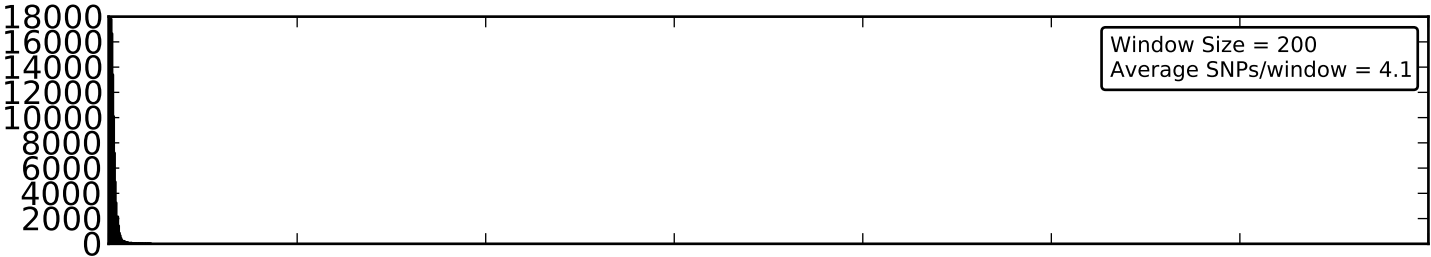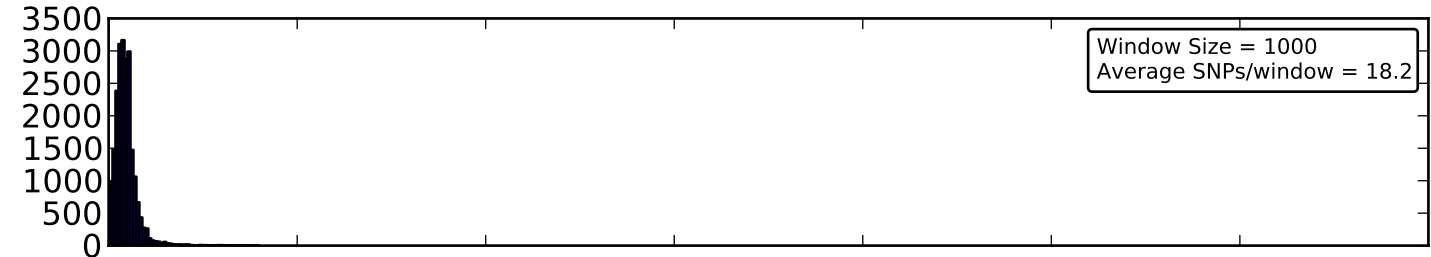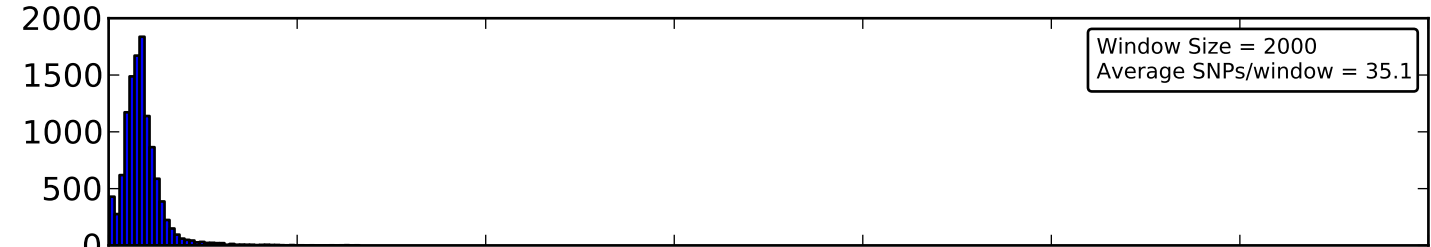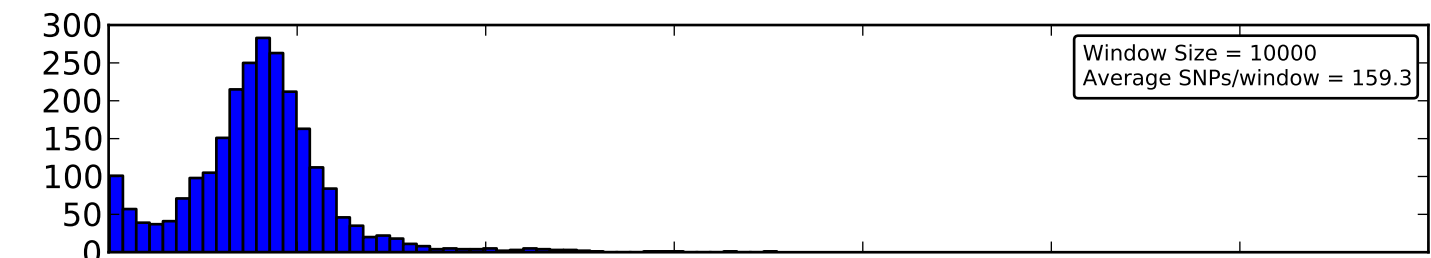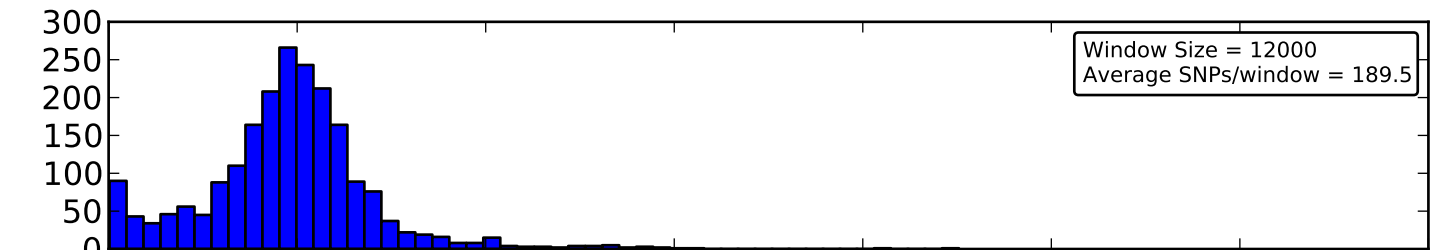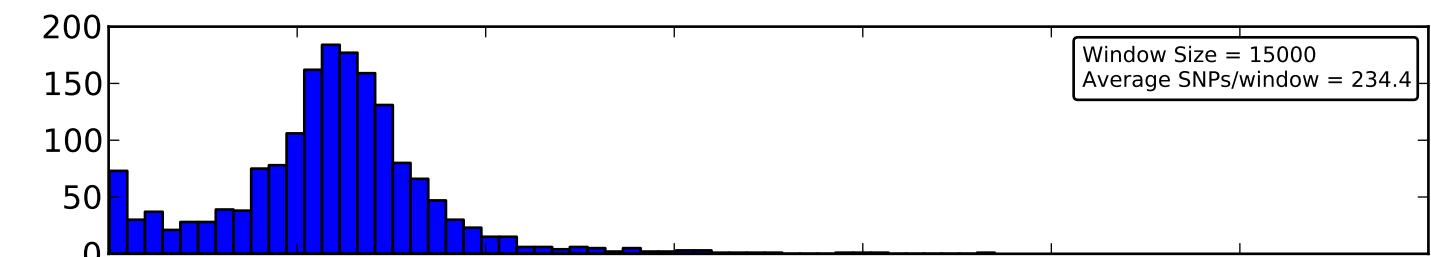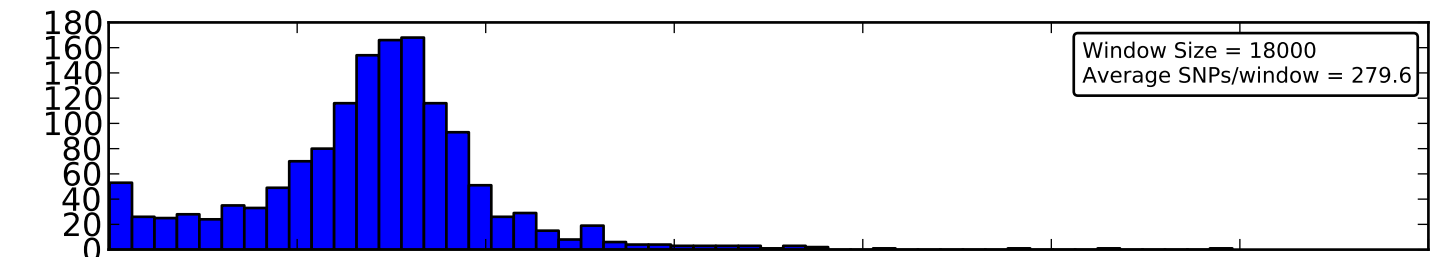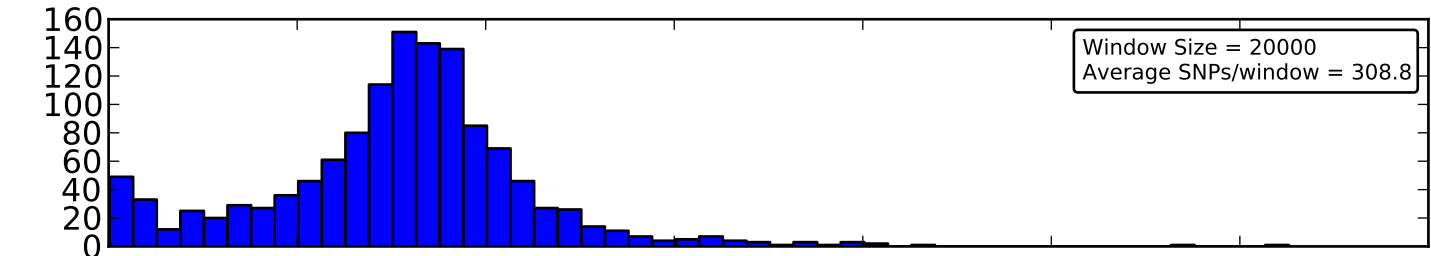

0 200 400 600 800 1000 1200 1400

Supplement: Supplementary Data [file supp_evu222_suppl_data.zip › SupplementaryFig1.pdf]

Distributions of Tajima's D, varying window size

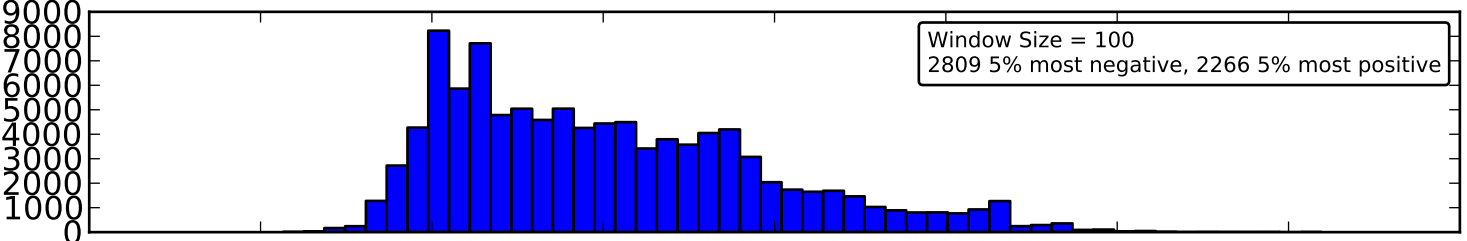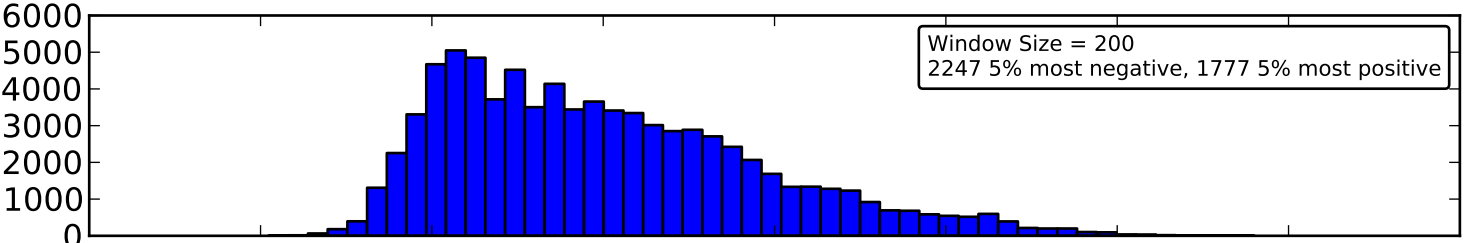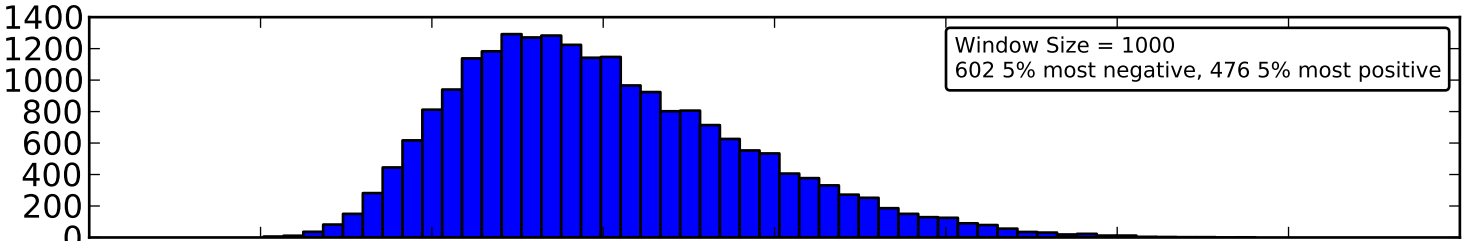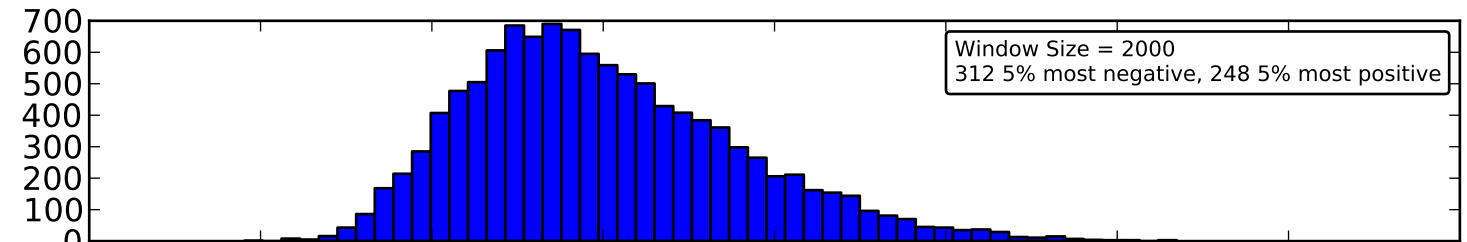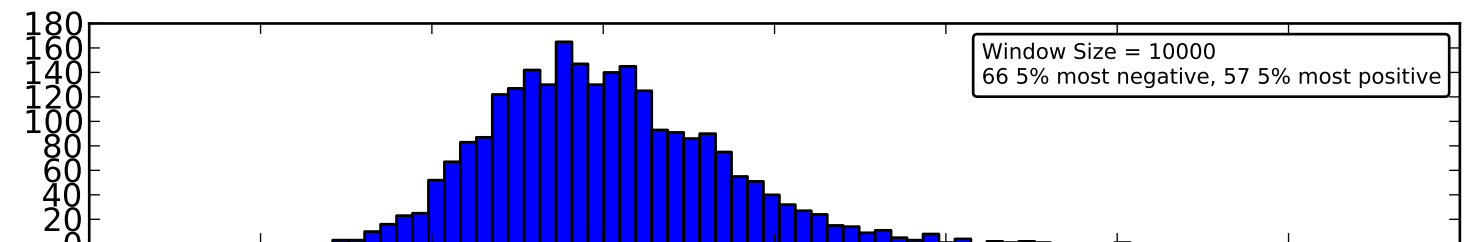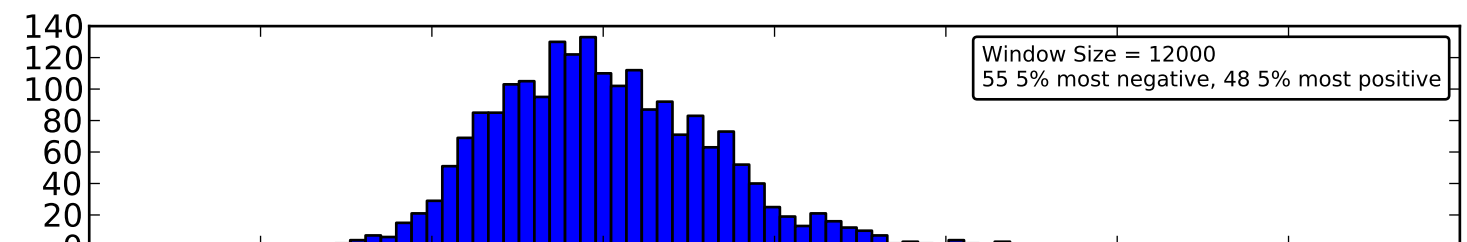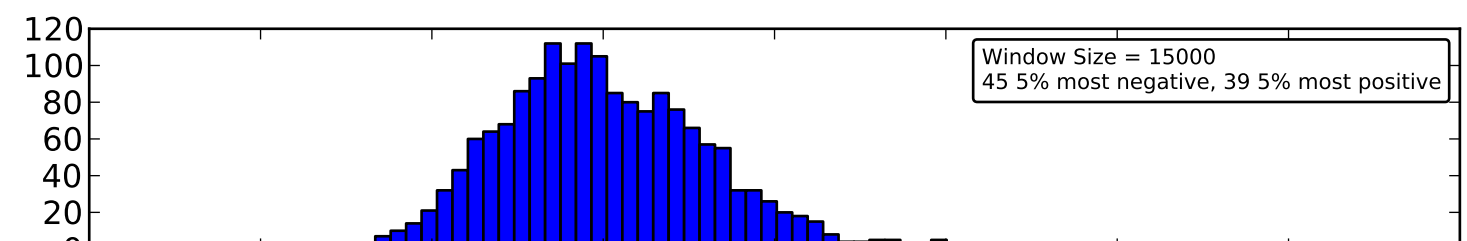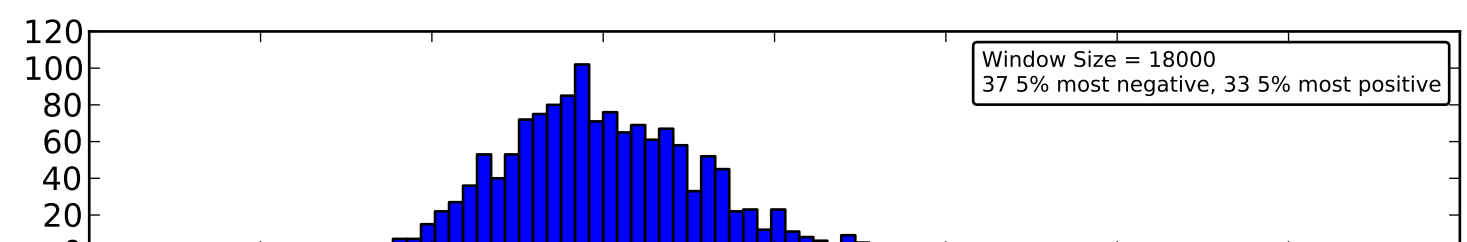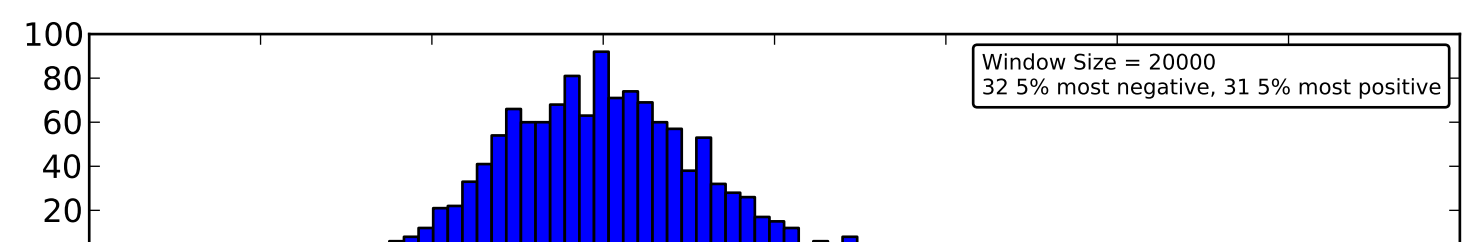

Supplement: Supplementary Data [file supp_evu222_suppl_data.zip › SupplementaryFig2.pdf]

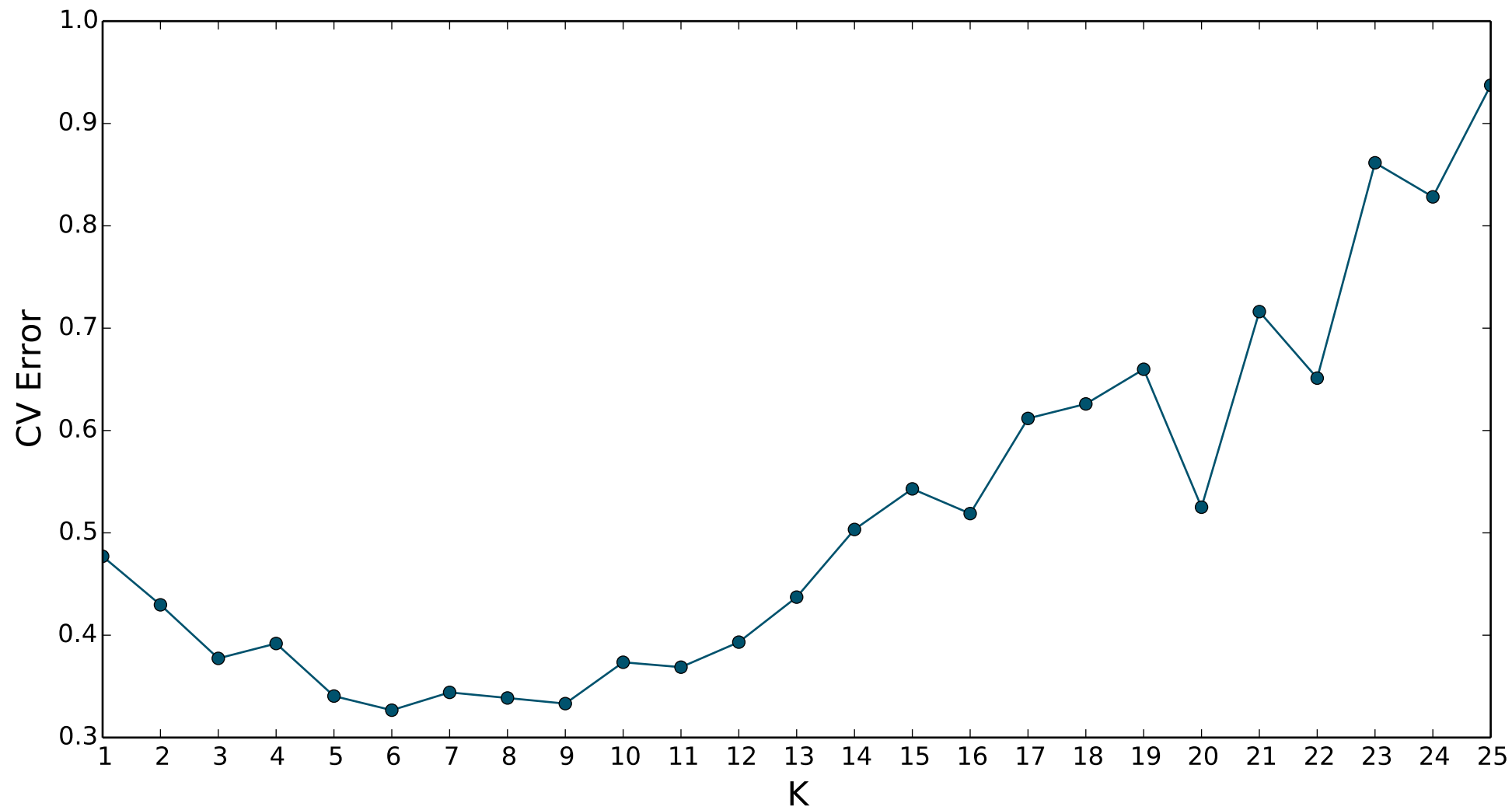

Supplement: Supplementary Data [file supp_evu222_suppl_data.zip › SupplementaryFig3.pdf]
