# Supplementary material for: Comparative Genomics Reveals Multiple Genetic Backgrounds of Human Pathogenicity in the Trypanosoma brucei Complex
Source: Genome Biol Evol. 2014 Oct 5;6(10):2811–9. doi: 10.1093/gbe/evu222 (PMC4224348; doi:10.1093/gbe/evu222)
Supplement: Supplementary Data [file supp_evu222_suppl_data.zip › Appendix 1.doc]

**Appendix 1:** Sample information for isolates. The first column shows the samples abbreviations. Sample names are made up of taxon designation (B-*Tbb*; E-*Te;* G1/2-*Tbg1/2*; R-*Tbr*), followed by one letter codes representing country of origin and host species, two digits representing the date of collection and in cases where two strains are identical to this point, a single separating digit. The second columns reports the isolate name according to the cryobank of origin. The next four columns provide information on the isolates taxonomy, geographic origin, host, and date of collection. The last four columns list the cluster to which is sampled is assigned to based on the Admixture analysis (Alexander et al. 2009) and information on the quality of the genomic data (number of read, coverage, and percent of reads mapped)

**Appendix 2:** A list of annotated genes found to be in windows for which a Tajima’s D score was in the highest 95% above and below zero, including the gene identification code (Gene ID), the, type of annotation, the number of SNPs present in the gene (no. SNPs), Tajima’s D score, the direction of selection (D =diversifying, P = purifying), the alpha value for the McDonald–Kreitman test (MK), where applicable, and the description of the annotation (Gene name)..

**Appendix 3:** Results of McDonald–Kreitman (MK) tests of selection for the 208 genes for which values could be obtained, including the gene identification code (Gene ID), the number of non-synonymous and synonymous sites (P_nonsyn and P_syn, respectively), the number of fixed non-synonymous and synonymous sites (D_nonsyn and D_syn, respectively), the neutrality index, and alpha score.

**Supplementary Figure 1:** Distribution of SNP densities in windows of 100, 200, 1000, 2000, 10,000, 12,000, 15,000, 18,000 and 20,000 base pairs across the 11 megabase chromosomes of the nuclear genome. Number of SNPs per window is indicated by the X axis, and the number of windows is indicated on the Y axis.

**Supplementary Figure 2:** Distribution of Tajima’s d values calculated in windows of 100, 200, 1000, 2000, 10,000, 12,000, 15,000, 18,000 and 20,000 base pairs across the 11 megabase chromosomes of the nuclear genome. Number of windows is indicated by the X axis, and the Tajima’s D score is indicated on the Y axis.

**Supplementary Figure 3:** A plot of cross validation error scores obtained from Admixture for clusters ranging from K=1 to 25. While scores for K=5-9 were similar, the lowest value was obtained for K=9. The value of K tested is indicated by the X axis and the cross validation score is indicated by the y axis.
